# Mental health problems in children and young people in Dutch general practice: trends in incidence and consultation rates from 2016 to 2022

**DOI:** 10.1007/s00127-025-02956-7

**Published:** 2025-07-18

**Authors:** Vincent R. ‘t Hart, Lukas B. M. Koet, Boris W. V. Schouten, Premysl Velek, Patrick J. E. Bindels, Heike Gerger

**Affiliations:** 1 Department of General Practice, Erasmus MC, University Medical Centre Rotterdam, PO Box 2040, Rotterdam, 3000 CA The Netherlands; 2 Department of Clinical Psychology, Open University, Heerlen, The Netherlands

**Keywords:** Child, Young people, Mental health, General practice

## Abstract

**Purpose:**

In recent decades, the prevalence of mental health problems among children and young people (CYP) has increased. It is unclear whether this increase in prevalence has also led to changes in health care utilization for these problems in general practice (GP). We therefore investigated time trends in incidence and consultation rates for eight mental health problems in CYP in Dutch general practice.

**Methods:**

We conducted a longitudinal population-based study using a GP-database (Rijnmond Primary Care Database) between 2016 and 2022. We extracted monthly data on mental health problems in CYP (0–24 years) in general practice. Using negative binomial models, we calculated trends for GP-registered incidence and consultation rates for different age and sex categories for the complete study period and for the period before the COVID-19 pandemic.

**Results:**

Consultation rates of all eight mental health problems increased significantly over time. Additionally, incidence rates for attention deficit hyperactivity disorder and depressive problems in both sexes, and anxiety problems in females increased significantly. Although we observed a decrease in incidence and consultations in the first months of the COVID-19 pandemic, overall trends did not differ from pre-pandemic trends.

**Conclusion:**

Our findings suggest an increasing workload for GPs for mental health problems in CYP. These observations imply the need for policymakers and GP-councils to develop new strategies which deal with these trends to ensure appropriate support and resources in the future.

**Supplementary Information:**

The online version contains supplementary material available at 10.1007/s00127-025-02956-7.

## Introduction

In recent decades, there has been a worldwide rise in the prevalence of mental health problems among children and young people (CYP) and associated health care use [1–4]. Although these increases are probably partially explained by more awareness of mental health and changes in help-seeking behavior, an actual decline in the mental health of CYP seems plausible [5]. Mental health problems in CYP are associated with significant short- and long-term negative effects including impaired quality of life, reduced workforce participation later in life and increased health care costs [6–9]. To prevent these negative impacts, early recognition and intervention is essential [10]. Unfortunately, mental health services have been under strain for years with long waiting lists due to a shortage of resources and professionals [11]. There are indications that the COVID-19 pandemic has led to worsening of mental health in CYP with increased demand for mental health care [12–14]. In the Netherlands, the mental health of CYP appears to have declined as well in recent years [15, 16]. Concurrently, the number of Dutch CYP receiving mental health care has risen [17, 18]. Although exact data are unavailable, waiting times for Dutch youth mental health services are considerable.

In the Netherlands, general practitioners (GPs) play a central role in primary care for CYP [19]. CYP seeking help for a mental health problem often first contact their GP for help. GPs can either manage these problems within their general practice or refer to specialized mental health care if needed. Since 2008 the function of the mental health practice nurse (MHPN) has been introduced to assist GPs in the management of (mainly adult) patients with mental health problems within general practice [20]. In 2016 approximately 90% of Dutch General practices employed a MHPN [21]. Since 2015 youth mental health practice nurses (YMHPN) focusing on children (< 18 years) have gradually been introduced into general practice [22]. There is no precise data on the number of YMHPNs working in general practice. However, in 2021, YMHPNs accounted for approximately 0.5% of the total hours worked in general practice [23]. Previous studies from Norway, Finland, and the Netherlands suggest that general practitioners (GPs) are increasingly consulted for mental health issues in children and young adults [12, 13, 24]. 

In an earlier study we found that consultation rates in CYP for mental health problems in general increased steadily between 2016 and 2021, with a similar trend before and during the COVID-19 pandemic [24]. It remains uncertain which specific mental health problems contributed to the observed increase in consultation rates, and whether trends varied among different types of mental health problems. In the present study, we therefore investigated time trends for eight mental health problems in CYP. We studied the rates and trends of GP-registered incidence and GP consultations for these problems in different sex and age groups. Additionally, we explored whether these trends were influenced by the COVID-19 pandemic.

## Methods

### Data sources and study population

We performed a longitudinal population-based study of CYP (aged 0–24 years) using the Rijnmond Primary Care Database (RPCD) data from January 2016 to December 2022. The RPCD is a region-specific derivative of the Integrated Primary Care Information database covering the Rotterdam metropolitan area [25]. Rotterdam is the second largest city of the Netherlands, with a large community of ethnic minorities and a comparatively high percentage of a low-income households [26]. Currently, more than half of the CYP living in Rotterdam are registered in a practice participating in the RPCD. Participating practices are located in the various neighborhoods of Rotterdam including socially deprived and non-deprived areas.

The RPCD contains pseudonymized medical data of general practice patients, including diagnostic codes, laboratory findings, prescriptions, referrals and specialists’ letters. Dutch GPs use the International Classification for Primary Care (ICPC-1) to register diagnoses, symptoms, and reasons for consultation [27]. For each patient, a categorical index of social deprivation (socially deprived area / socially non-deprived area) was available based on the postal code of their residence. This index, defined by the Dutch Healthcare Authority, is derived from area characteristics such as the number of social benefit recipients, residents with low income, and non-Western immigrants [28]. The RPCD database has extensive quality control measures [25]. 

### Outcomes

We investigated the occurrence of attention deficit hyperactivity disorder (ADHD), anxiety problems, behavioural problems, depressive problems, eating disorders (i.e. anorexia nervosa and bulimia), sleeping problems, substance abuse, and suicidality (i.e. suicidal ideations, attempts and suicide). These mental health problems were chosen for investigation because they have clearly defined ICPC codes and are either relatively common or very relevant mental health problems. For each mental health problem, we grouped the relevant ICPC codes, including both symptom and diagnosis codes (e.g., P01 “Feeling anxious/nervous/tense” and P74 “Anxiety disorder” for anxiety problems). Supplementary eTable 1 provides an overview of the ICPC codes used for each mental health problem.

Although GPs registered the ICPC codes in patients’ records, it is unclear whether these codes were based on their own assessments or diagnoses provided by mental health specialists. It is also important to note, that GPs often do not apply formalized diagnostic procedures for assessing the presence of mental health disorders. Therefore, the investigated mental health problems may not necessarily meet the DSM-5 diagnostic criteria. This approach reflects daily GP practice, where GPs encounter a wide range of mental health problems, from mild to severe.

We used two outcomes for our analysis: incidence rates and consultation rates. We calculated GP-registered incidence rates per month and per year for each mental health problem. A new incidence was defined as the first time a GP registered a specific mental health problem in a patient’s medical file. If a patient received a new relevant ICPC code but had previously been registered with an ICPC code from the same problem category, it was not considered a new incidence (e.g., if a patient previously received the ICPC code P01 “Feeling anxious/nervous/tense” and later received P74 “Anxiety disorder,” only the first coding of P01 was considered a new incidence). To calculate monthly incidence rates, we divided the number of new cases per mental health problem (numerator) by the number of at-risk CYP (denominator) each month. The at-risk population excluded CYP previously diagnosed with the specific mental health problem. This was done separately for each mental health problem (e.g., once a child was diagnosed with an anxiety problem, they were removed from the population at risk for anxiety problems but remained at risk for other mental health problems).

We calculated consultation rates per problem category. A consultation was defined as any contact (in person, by phone, or online) with a relevant ICPC code between a GP and a patient or a GP and a specialist (including specialist letters) where symptoms, diagnosis, or treatment were discussed. For monthly consultation rates, we extracted all relevant contacts (numerator) for each mental health problem and divided them by the number of registered CYP per month (denominator).

Yearly rates were calculated by aggregating the monthly rates. We calculated incidence and consultation rates for individual mental health problems for all included patients and subgroups based on sex and four age groups: young children (0–6 years), primary school children (7–12 years), adolescents (13–17 years), and young adults (18–24 years).

### Data-analysis

Analyses were performed using R (version 4.3.1, MASS package) [29]. To assess time trends, we fitted negative binomial regression models to the monthly counts of first diagnoses and consultations for each mental health problem over the entire study period (2016–2022).

In each model, we tested for linear trends over time by including a continuous covariate representing the number of months since the start of the study. We also included a categorical variable for calendar month to adjust for seasonal variations (e.g., reductions during summer holidays). The primary variable of interest was the linear trend over time, which we presented as the relative rate (RR) per incremental month.

We repeated the analyses per sex and age group. In models without significant overdispersion, we used Poisson regression. We used autocorrelation-plots to detect possible autocorrelation. In case of autocorrelation, we corrected for this by including first-order lagged residuals. Given the exploratory nature of this study, adjustments for multiple testing were not made.

### Sensitivity analyses

We identified two potential effect modifiers, the COVID-19 pandemic period and a slight increase of CYP living in social deprivation areas from fall 2020 onwards. To assess influence of these two factors we conducted sensitivity analyses using the pre-pandemic period (until March 2020), in which the percentage of CYP living in social deprivation areas was stable. For this period, we fitted negative binomial regression models per mental health problem for the total sample. These were used to predict incidence and consultation rates during and after the pandemic. Firstly, we plotted the observed and expected incidence and consultation rates with their 95% confidence intervals (95%CI) (supplementary eFigure 1) per mental health problem and visually compared these for differences. Secondly, we calculated the expected number of new incidences and consultations for the COVID-19 period (March 2020-April 2022) by adding up the expected monthly rates with their 95%CI and compared these with the observed numbers. Thirdly, to assess changes in trends due to the pandemic, we compared the time trends for the complete study period (January 2016–December 2022) with pre-pandemic trends (January 2016–February 2020).

### Ethics and data availability

The study was approved by the Governance Board of the RPCD (project-number 2020.012). Patient data was pseudonymized. Therefore, by Dutch law, no patient consent is required. We followed the RECORD guidelines [30]. Due to legal constraints, data is not publicly available, and access requires approval by the RPCD Governance Board.

## Results

### Cohort characteristics

The number of CYP in the RPCD increased from 60,892 patients in January 2016 to 105,103 in December 2022. In total, 49.2% was female and mean age was 12.6 (SD 6.1) years. The percentage of CYP living in socially deprived areas increased from 18.2 to 21.7% over time. Changes in cohort demographics can be explained by more practices joining the RPCD. Cohort demographics are presented in Table 1.

### Incidence rates per year

**Fig. 1:**
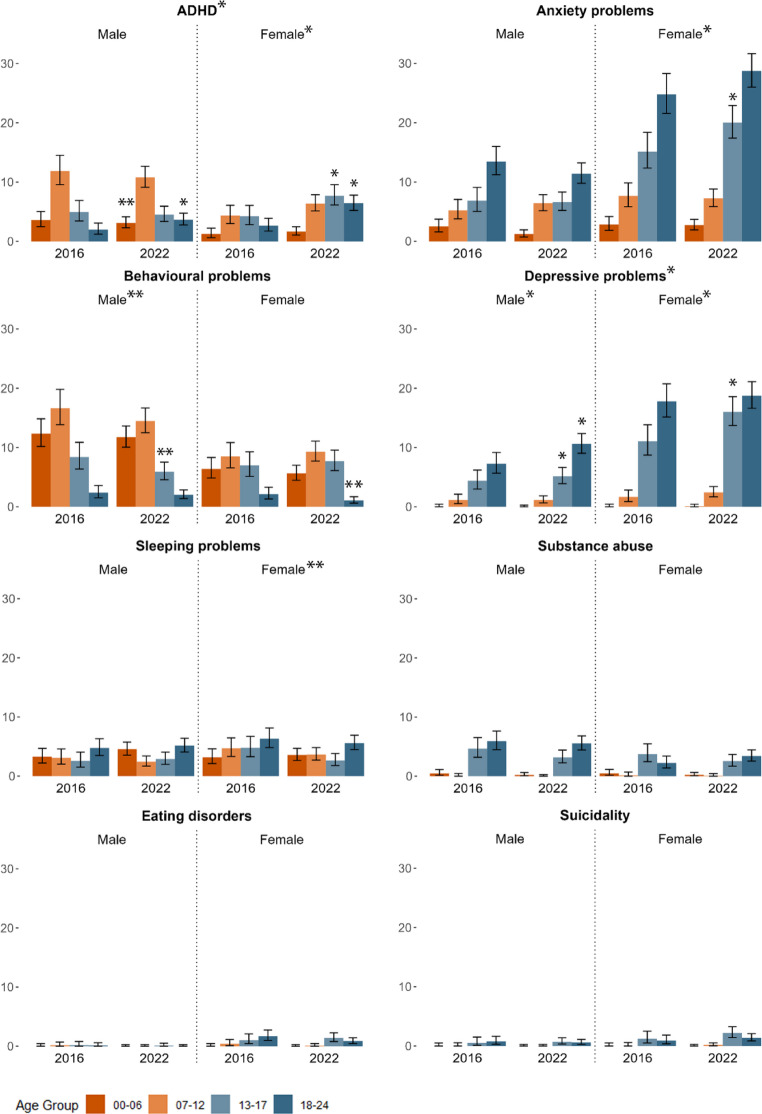
Incidence per sex and age group (in 1000 person-years) in 2016 and 2022 with 95% confidence interval. *significant increasing (modelled) monthly trend over (sub)group. ** significant decreasing (modelled) monthly trend over (sub)group

The incidence rates per mental health problem for the years 2016 and 2022 are presented in Fig. 1 and Table 2 (see supplementary eTable 2 and 3 for details per age and sex per year). Overall, highest incidences were found for anxiety problems, ADHD and depressive problems.

**Table 1:** Cohort size of children and young people (0–24) measured in July each year

Year	Dynamic cohort size	Females	Mean age (SD)	Living in deprived area
2016	68,599	49.2%	12.60 (7.09)	18.2%
2017	76,644	49.1%	12.64 (7.09)	17.9%
2018	78,424	49.0%	12.65 (7.09)	18.0%
2019	79,287	48.9%	12.67 (7.08)	17.8%
2020	96,044	49.0%	12.58 (7.04)	18.6%
2021	109,754	49.0%	12.57 (7.03)	21.9%
2022	112,845	49.0%	12.59 (7.03)	21.7%

Due to increasing number of participating practices in the Rijnmond primary care database during study period, more patients were added to the dynamic cohort over time

**Table 2:** Average incidence and consultations per year. Average incidence rate and consultation rate per year (per 1000 person-years) of investigated mental health problems with 95% confidence intervals

	Incidence per 1000 person-years		Consultations per 1000 person-years	
	2016	2022	2016	2022
ADHD	4.15 (3.68–4.67)	5.42 (4.99–5.87)	41.33 (39.82–42.89)	55.88 (54.51–57.29)
Anxiety problems	9.76 (9.02–10.54)	10.30 (9.70–10.92)	42.48 (40.94–44.05)	70.01 (68.47–71.58)
Behavioral problems	7.70 (7.05–8.40)	7.05 (6.56–7.57)	29.96 (28.67–31.29)	41.73 (40.54–42.94)
Depressive problems	5.38 (4.84–5.97)	6.57 (6.10–7.07)	37.16 (35.72–38.64)	60.71 (59.28–62.17)
Sleeping problems	4.12 (3.65–4.64)	3.91 (3.54–4.29)	9.79 (9.06–10.56)	11.31 (10.69–11.95)
Substance abuse	2.16 (1.82–2.54)	1.91 (1.66–2.18)	9.55 (8.83–10.31)	9.89 (9.31–10.49)
Eating disorders	0.43 (0.29–0.61)	0.27 (0.18–0.38)	3.23 (2.82–3.96)	4.51 (4.13–4.93)
Suicidality	0.41 (0.26–0.61)	0.58 (0.44–0.74)	1.22 (0.96–1.53)	3.05 (2.73–3.39)

### Time trends in incidence rates

Time trends per mental health problem are presented in Table 3. Supplementary eTable 4 shows the results per age and sex category. No or too few incident cases for allowing subgroup analyses were seen in the following subgroups: eating disorders in male subgroup across all age categories and in females aged 0–12; depressive problems in both sexes aged 0–6; substance abuse and suicidality in both sexes aged 0–12 (see supplementary eTable 4). We detected no autocorrelation in any of the models.

**Table 3:** Time trends in incidence and consultations for investigated mental health problems

	Time trends in incidence		Time trends in consultations	
	Monthly relative rate	*p*-value*	Monthly relative rate	*p*-value*
ADHD	1.004 (1.002–1.006)	<0.001	1.005 (1.004–1.006)	<0.001
Anxiety problems	1.001 (1.000–1.002)	0.164	1.008 (1.007–1.009)	<0.001
Behavioral problems	0.999 (0.997–1.000)	0.106	1.005 (1.004–1.006)	<0.001
Depressive problems	1.003 (1.001–1.005)	0.004	1.008 (1.007–1.009)	<0.001
Sleeping problems	0.999 (0.997–1.000)	0.095	1.002 (1.001–1.004)	0.001
Substance abuse	0.999 (0.997–1.001)	0.400	1.002 (1.001–1.004)	0.007
Eating disorders	1.000 (0.994–1.005)	0.945	1.007 (1.005–1.010)	<0.001
Suicidality	1.004 (0.999–1.009)	0.122	1.013 (1.009–1.016)	<0.001

Monthly trends in relative rate with 95% confidence interval per mental health problem

* Given the exploratory nature of this study, p-values are not adjusted for multiple testing

For ADHD, the monthly incidence rate increased significantly over time (RR = 1.004; 95%CI 1.002–1.006). The steepest trends were seen in adolescent girls aged 13–17 (RR = 1.012; 95%CI 1.007–1.018) and young women aged 18–24 (RR = 1.017; 95%CI 1.011–1.022). The monthly incidence rate of depressive problems increased significantly over time (RR = 1.003; 95%CI 1.001–1.005). This trend was most pronounced in children aged 13–17 (boys: RR = 1.005; 95%CI 1.000-1.009, girls: RR = 1.005; 95%CI 1.002–1.008). Anxiety problems had no significant trend in the total sample, but we observed a significantly increasing trend in females (RR = 1.002; 95CI% 1.001–1.004) which was mainly explained by the increasing trend in girls aged 13–17 (RR = 1.005; 95%CI 1.002–1.008). Sleeping problems showed a significantly negative trend among females (RR = 0.998; 95%CI 0.995-1.000) as did behavioural problems in the overall male subgroup (RR = 0.998; 95%CI 0.996-1.000) and female subgroup aged 18–24 (RR = 0.965; 95%CI 0.946–0.984). There were no significant trends in incidence for eating disorders (RR = 1.000; 95%CI 0.994–1.005), substance abuse (RR = 0.999; 95%CI 0.997–1.001) or suicidality (RR = 1.004 95%CI 0.999–1.009).

### GP consultation rates

**Fig. 2:**
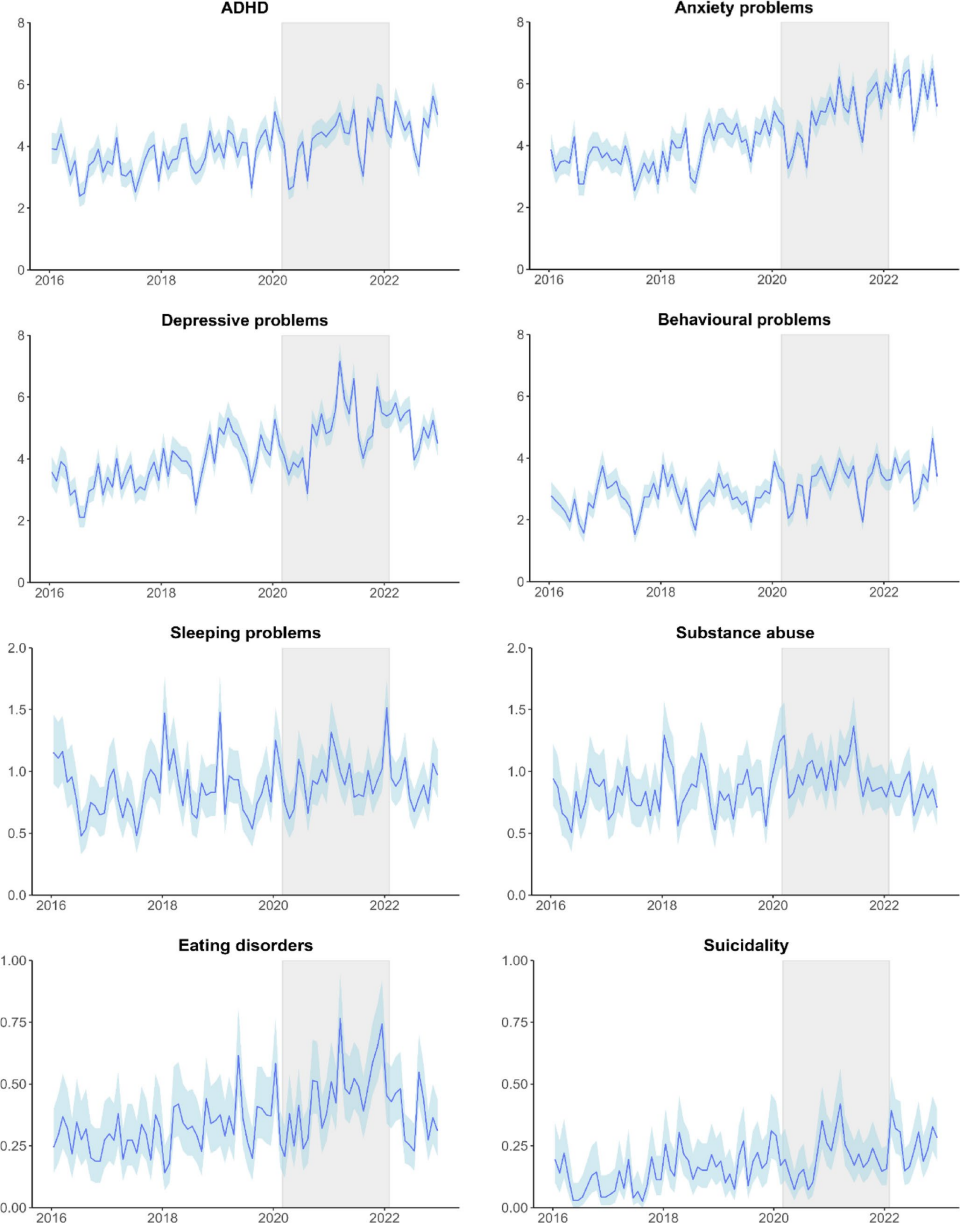
Monthly consultation rates over time. This figure displays the monthly consultation rates per 1,000 person-months over time, along with 95% confidence intervals. The COVID-19 period (March 2020 to April 2022) is highlighted in grey

Yearly consultation rates in 2016 and 2022 are presented in Table 2. The highest consultation rates were found for anxiety problems, ADHD and depressive problems. Figure 2 shows monthly consultation rates from 2016 to 2022.

### Time trends in GP consultation rates

Overall consultation rates increased significantly for all mental health problems between 2016 and 2022 (Table 3). Supplementary eTable 5 shows the results per age and sex category. No or too few consultations for allowing subgroup analyses were seen for substance abuse, suicidality and eating disorders in both sexes aged 0–12 and for depressive disorders in both sexes aged 0–6. Although we detected autocorrelation in some models, correction for autocorrelation did not change our results (see supplementary eTable 6 for corrected estimates).

For anxiety problems the largest increasing trend was found in girls aged 13–17 (RR = 1.013; 95%CI 1.011–1.015). For ADHD, monthly consultation rates showed a significantly increasing trend over time, which was mainly explained by increases in consultations in adolescents and young adults of both sexes. The steepest trend was seen in young women aged 18–24 (RR = 1.013; 95%CI 1.011–1.015). We found a negative trend in males aged 0–6 (RR = 0.994; 95%CI 0.990–0.997). Trends in depressive problems were significantly increasing and similar for males (RR = 1.008; 95%CI 1.007–1.010) and females (RR = 1.007; 95%CI 1.006–1.009). Suicidality-related consultations showed the strongest increasing trend over time (RR = 1.013; 95%CI 1.009–1.016) in the total sample. This should be interpreted with caution due to the low number of consultations (1.22 consultations/1000 person-years in 2022), as few extra consultations could affect the trend significantly. Consultation rates of eating disorders remained stable in males (RR = 1.000; 95%CI 0.989–1.011) and significantly increased in females (RR = 1.008; 95%CI 1.005–1.011). There was a decreasing trend in males aged 18–24 (RR = 0.965; 95%CI 0.946–0.984). Consultation rates for behavioural problems increased significantly for both males (RR = 1.004; 95%CI 1.003–1.005) and females (RR = 1.005; 95%CI 1.004–1.007). There was a significant increasing trend in sleeping problems particularly among young women (RR = 1.005; 95%CI 1.003–1.007). In contrast, while overall consultation rates related to substance abuse increased (RR = 1.002; 95%CI 1.001–1.004), there was a decrease in children aged 13–17 (boys: RR = 0.996; 95%CI 0.992–0.999; girls RR = 0.994; 95%CI 0.988–0.999).

### Sensitivity analyses

In the first months of the pandemic (March-May 2020), incidence and consultation rates for most mental health problems were lower than expected. Hereafter, the rates returned to expected levels based on pre-pandemic trends (supplementary eFigure 1, eTables 7–8). In general, there were no or small differences between the observed trend before the pandemic and during the entire study period.

Sleeping problems incidence, however, showed a significant difference with an increasing trend pre-COVID and a non-significant negative trend in the entire study period (pre-COVID: RR = 1.006; 95%CI 1.003–1.009, entire study period: RR = 0.999; 95%CI 0.997-1.000). The observed cases were within the 95%CI of expected cases (observed cases: 878; expected cases: 1023; 95%CI 814–1286).

The increasing trend in incidence rate for depressive problems was more pronounced before the COVID-19 pandemic compared to the trend over the entire study period (pre-pandemic: RR = 1.006; 95%CI: 1.003–1.009, entire study period: RR = 1.003; 95%CI: 1.001–1.005). However, this did not result in significant differences between observed and expected incident cases (observed 1483; expected: 1700; 95%CI 1398–2067) during the pandemic period.

Increasing trends in consultation rates for suicidality problems seemed somewhat higher before the pandemic when compared with the entire study period (pre-pandemic: RR = 1.019 95%CI 1.011–1.028 entire study period: RR = 1.013 95CI% 1.009–1.016). The observed number of consultations, however, was still within the 95%CI (observed: 565; expected: 770; 95%CI 462–1283).

## Discussion

### Main findings

In this study, we analyzed trends in mental health problems among children and young people in general practice from 2016 to 2022. The three most common mental health problems in our cohort were anxiety problems, ADHD, and depressive problems. When examining trends in GP-registered incidence across the entire study population, we observed increasing trends over time for ADHD and depression. Subgroup analyses by age and sex mostly showed stable or increasing trends, with some decreasing trends as well. GP consultation rates for all investigated mental health problems increased significantly across the entire study population. The most notable increases were observed for anxiety and depressive problems. Additionally, we found relevant differences in trends between age and sex categories for some mental health problems. Overall, COVID-19 did not have a significant effect on observed trends over time: after an initial decrease during the first months of the pandemic, both incidence and consultation rates showed similar trends compared with those observed pre-pandemic. Furthermore, the observed number of incident cases and consultations fell within the 95% confidence intervals of expected values.

### What is already known on this topic

Our study aligns with previous reports indicating increasing trends in mental health problems among CYP [16, 31, 32]. During 2004–2008 increases in the prevalence of anxiety, depression, and alcohol abuse were reported in Dutch general practices [16]. Increasing trends (1995–2014) and high prevalence of mental health problems were also seen in other European countries with prevalence rates up to 15.5% [31, 32]. It has been suggested that one reason for these increasing trends might be greater awareness of mental health problems among professionals and patients [5]. For example, in recent years there is more information about sex-differences in the presentation of ADHD [33, 34]. This increased awareness among professionals may have led to improved recognition and increased incidence rates in females.

Depressive and anxiety disorders in CYP have seen increasing trends before 2020 in Europe, the USA and Asia [36–39]. Although several studies reported that the prevalence of depressive and anxiety symptoms increased during the COVID-19 pandemic, the exact impact of the pandemic on these problems is debated [39, 40]. 

Several studies using primary care registry data reported higher numbers of mental health-related GP-consultations in CYP during the pandemic, compared with the period before the pandemic [12, 13, 41, 42]. Significant increases were mainly seen in consultations related to anxiety, mood and eating disorders [12, 41, 42]. Only a limited number of studies used longitudinal trends before and during the pandemic [13, 41, 42]. 

### What this study adds

In our study, consultation rates increased strongly across all mental health problems suggesting increased health care utilization in general practice. It is therefore plausible that the previously observed increase in consultation rates [12, 13, 24] is due to a rise in demand for care for various psychological problems rather than one specific mental health problem. However, the number of consultations for anxiety and depressive problems increased strongest over time and may largely explain the observed overall increase. As GP consultations for mental health problems in CYP are increasing over time, the role of GPs in the management of these problems may have become more important, accompanied by a higher workload for GPs.

In interpreting our results, it is important to note that some RPCD practices began employing a YMHPN during the final years of our study period. Due to database properties and pseudonymization, we cannot determine the exact number of practices with a YMHPN, nor can we differentiate between consultations with a GP and those with a practice nurse. Part of the observed increase in consultations may be due to the enhanced care capacity within general practice resulting from the availability of YMHPNs. However, we estimate the impact of YMHPNs on our results to be small. In the Rotterdam metropolitan area, only a minor portion of practices started employing a YMHPN, and they were active only in the last years of the study period.

Whereas earlier studies suggested that the COVID-19 pandemic led to increased consultations for mental health problems in CYP, the observed trends in our study existed before and continued throughout the pandemic. This steady increase over time suggests that the COVID-19 pandemic had limited impact on help-seeking behaviour for mental health problems in our study population. Based on the steady increasing trends, we speculate that GPs will continue to experience an increasing demand for mental healthcare for CYP in the coming years.

The main strength of this study is that it reports a comprehensive overview of trends in both incidence and consultations rates of mental health problems in CYP in general practice. Contrary to many other studies using cross-sectional samples, we used longitudinal data from seven consecutive years, covering the complete COVID-19 pandemic. Due to this longitudinal design, we were able to demonstrate existing upward trends while correcting for seasonality and taking possible effects of the pandemic into account.

Future studies should focus on investigating possible factors causing these increasing trends, whether it be increasing awareness, increasing help-seeking behaviour, waiting times for secondary care, transfer of care from specialized mental health care to general practice or actual increases in the occurrence of mental health problems in CYP. Additionally, we recommend that policymakers and medical councils proactively develop strategies to address these trends and ensure appropriate support and resources are available in primary care settings.

### Limitations

First, the RPCD data only represent cases of mental health problems which are documented within the GP patient file. In the Netherlands GPs have a comprehensive patient file (which includes specialists’ letters). The completeness of this file depends on other professionals providing information to the GP. GP-registered incidence may therefore underestimate the overall incidence. Second, the RPCD consists of health care data not primarily designed for research purposes. Therefore, misclassification can occur. Third, due to limitations of the database, we cannot differentiate between consultations with a GP and with a practice nurse. As such, it remains unclear to what extend the observed increases in consultations are to be attributed to the presence of YMHPNs in general practice. Fourth, it was unclear from our data what proportion of the mental health problems reflected a specific mental health disorder diagnosed by a GP or a mental health specialist. Many GPs do not use standardized diagnostic procedures. Consequently, the ICPC-coded mental health problems will frequently not meet the DSM-5 criteria for a mental health disorder. Fifth, due to the dynamic cohort design, the percentage of CYP living in deprived areas in our cohort increased slightly over time. This could in theory have influenced the observed trends in incidence and consultation rates. In our sensitivity analysis of the pre-pandemic trends, the percentage of CYP living in deprived areas was stable and the results in that study period did not differ significantly from our main results covering the entire study period. Moreover, previous research in Rotterdam reported similar help-seeking behaviour for mental health problems in children between neighbourhoods with different social economic status [43]. Therefore, we consider the risk that the observed slight change in demographics of the study population over time significantly influenced our findings to be small. Sixth, despite our large dataset, the trends for rare problems (e.g., suicidality and eating disorders) should be interpreted with caution, as a minor increase in cases may affect the overall trend. Seventh, it is important to recognize that our study focused on trends and rates of mental health problems in general practice, which are dependent on the actual help-seeking behaviour of patients. Therefore, our results should be interpreted in conjunction with results from other study types (e.g., prevalence studies).

## Conclusion

GPs in our cohort have seen a steadily increasing trend in consultation rates for all eight investigated mental health problems in CYP, which started well before the COVID-19 pandemic. Incidence increased for certain problems. The increased utilization of GP care for mental health problems indicates higher workload and an increasingly important role of Dutch general practices for these patients. Based on the consistent trend over the observed seven years we assume that the increasing trend in consultations will continue into the near future. This increasing demand for mental health care requires the development of new strategies by policy makers and GP-councils to ensure adequate support and resources for these problems in the future.

## Electronic supplementary material

Below is the link to the electronic supplementary material.

Supplementary Material 1

## Data Availability

The study was approved by the Governance Board of the RPCD (project-number 2022.032). Patient data was pseudonymized. Therefore, by Dutch law, no patient consent is required. We followed the RECORD guidelines [30]. Due to legal constraints, data is not publicly available, and access requires approval by the RPCD Governance Board.
