# Supplementary material for: Mental health problems in children and young people in Dutch general practice: trends in incidence and consultation rates from 2016 to 2022
Source: Soc Psychiatry Psychiatr Epidemiol. 2025 Jul 18;61(1):29–39. doi: 10.1007/s00127-025-02956-7 (PMC12855347; doi:10.1007/s00127-025-02956-7)
Supplement: Supplementary file 1 — Supplementary Material 1 [file 127_2025_2956_MOESM1_ESM.docx]

Contents

[eTable 1 International Classification of Primary Care (ICPC) codes 2](#_Toc163580073)

[eTable 2 Males: incidence of mental health problems 3](file:///\\storage.erasmusmc.nl\m\MyDocs\307004\My%20Documents\Wetenschapsstage\08%20Review\2024-04-09\supplementary%20tables%20and%20figures.docx#_Toc163580074)

[eTable 3 Females: incidence of mental health problems 4](file:///\\storage.erasmusmc.nl\m\MyDocs\307004\My%20Documents\Wetenschapsstage\08%20Review\2024-04-09\supplementary%20tables%20and%20figures.docx#_Toc163580075)

[eTable 4 Monthly trends of incidence 5](file:///\\storage.erasmusmc.nl\m\MyDocs\307004\My%20Documents\Wetenschapsstage\08%20Review\2024-04-09\supplementary%20tables%20and%20figures.docx#_Toc163580076)

[eTable 5 Monthly trends of consultations 6](file:///\\storage.erasmusmc.nl\m\MyDocs\307004\My%20Documents\Wetenschapsstage\08%20Review\2024-04-09\supplementary%20tables%20and%20figures.docx#_Toc163580077)

[eTable 6 Monthly trends of consultation rates, after correction for autocorrelation 7](file:///\\storage.erasmusmc.nl\m\MyDocs\307004\My%20Documents\Wetenschapsstage\08%20Review\2024-04-09\supplementary%20tables%20and%20figures.docx#_Toc163580078)

[eTable 7 Monthly trends of incidence, before March 2020 and for 2016-2022 8](file:///\\storage.erasmusmc.nl\m\MyDocs\307004\My%20Documents\Wetenschapsstage\08%20Review\2024-04-09\supplementary%20tables%20and%20figures.docx#_Toc163580079)

[eTable 8 Monthly trends of consultation rates, before March 2020 and for 2016-2022 9](file:///\\storage.erasmusmc.nl\m\MyDocs\307004\My%20Documents\Wetenschapsstage\08%20Review\2024-04-09\supplementary%20tables%20and%20figures.docx#_Toc163580080)

[eFigure 1a modelled monthly incidence rate vs observed incidence 10](file:///\\storage.erasmusmc.nl\m\MyDocs\307004\My%20Documents\Wetenschapsstage\08%20Review\2024-04-09\supplementary%20tables%20and%20figures.docx#_Toc163580081)

[eFigure 1a modelled monthly incidence rate vs observed incidence - continued 11](file:///\\storage.erasmusmc.nl\m\MyDocs\307004\My%20Documents\Wetenschapsstage\08%20Review\2024-04-09\supplementary%20tables%20and%20figures.docx#_Toc163580082)

[eFigure 1b modelled monthly consultation rate vs observed consultation rate 12](file:///\\storage.erasmusmc.nl\m\MyDocs\307004\My%20Documents\Wetenschapsstage\08%20Review\2024-04-09\supplementary%20tables%20and%20figures.docx#_Toc163580083)

[eFigure 1b modelled monthly consultation rate vs observed consultation rate - continued 13](file:///\\storage.erasmusmc.nl\m\MyDocs\307004\My%20Documents\Wetenschapsstage\08%20Review\2024-04-09\supplementary%20tables%20and%20figures.docx#_Toc163580084)

## eTable 1 International Classification of Primary Care (ICPC) codes

| Problem category | ICPC code |
| --- | --- |
| ADHD | P21 (Overactive child, hyperkinetic) |
| Anxiety problems | P01 (Feeling anxious/nervous/tense), P74 (Anxiety disorder/anxiety state) |
| Behavioural problems | P04 (Feeling/behaving irritable/angry), P22 (Other concern with behaviour of child), P23 (Other symptoms/complaints concerning behaviour of adolescent) |
| Depressive problems | P03 (Feeling depressed), P76 (Depressive disorder) |
| Eating disorders (i.e. anorexia nervosa, boulemia) | T06 (Anorexia nervosa/bulemia) |
| Sleeping problems | P06 (Disturbances of sleep/insomnia) |
| Substance abuse | P15 (Chronic alcohol abuse),  P16 (Acute alcohol abuse),  P17 (P17 Tobacco abuse),  P18 (Medicinal abuse),  P19 (Drug abuse) |
| Suicidality | P77 (Suicide attempt/Suicide) |

##

| Males: Incidence per Problem (cases/1000 person years with 95% confidence intervals) Categorized by year and age category (years) eTable 2 Males: incidence of mental health problems | | | | | | | | | | |
| --- | --- | --- | --- | --- | --- | --- | --- | --- | --- | --- |
| **year** | **sex** | **age group** | **ADHD** | **Anxiety problems** | **Eating disorders** | **Behavioral problems** | **Depressive problems** | **Sleeping problems** | **Substance abuse** | **Suicidality** |
| 2016 | Male | 00-06 | 3.58 (2.46-5.03) | 2.49 (1.58-3.73) | 0 (0-0.4) | 12.33 (10.15-14.83) | 0 (0-0.40) | 3.27 (2.21-4.67) | 0.43 (0.12-1.10) | 0 (0-0.48) |
| 2017 | Male | 00-06 | 4.54 (3.34-6.04) | 1.93 (1.18-2.98) | 0.12 (0-0.64) | 10.88 (8.95-13.1) | 0 (0-0.35) | 5.15 (3.86-6.74) | 0.38 (0.10-0.98) | 0 (0-0.43) |
| 2018 | Male | 00-06 | 3.96 (2.85-5.35) | 1.03 (0.52-1.85) | 0.22 (0.03-0.81) | 11.21 (9.27-13.43) | 0 (0-0.35) | 4.56 (3.36-6.04) | 0.56 (0.21-1.23) | 0 (0-0.35) |
| 2019 | Male | 00-06 | 2.77 (1.87-3.95) | 1.56 (0.91-2.50) | 0 (0-0.45) | 11.72 (9.75-13.96) | 0 (0-0.34) | 3.44 (2.42-4.74) | 0.46 (0.15-1.07) | 0 (0-0.37) |
| 2020 | Male | 00-06 | 2.97 (2.09-4.09) | 1.52 (0.92-2.38) | 0 (0-0.44) | 8.88 (7.29-10.71) | 0.16 (0.02-0.58) | 3.88 (2.86-5.14) | 0.24 (0.05-0.70) | 0 (0-0.3) |
| 2021 | Male | 00-06 | 2.36 (1.65-3.29) | 1.48 (0.93-2.25) | 0 (0-0.25) | 10.62 (9.02-12.43) | 0.27 (0.07-0.69) | 4.35 (3.35-5.56) | 0.4 (0.15-0.88) | 0 (0-0.25) |
| 2022 | Male | 00-06 | 3.09 (2.26-4.12) | 1.21 (0.72-1.91) | 0 (0-0.25) | 11.73 (10.04-13.62) | 0 (0-0.25) | 4.54 (3.52-5.76) | 0.2 (0.04-0.59) | 0 (0-0.25) |
| 2016 | Male | 07-12 | 11.85 (9.55-14.53) | 5.22 (3.76-7.05) | 0.12 (0-0.68) | 16.63 (13.85-19.8) | 1.1 (0.5-2.09) | 3.09 (2-4.56) | 0 (0-0.45) | 0 (0-0.54) |
| 2017 | Male | 07-12 | 10.62 (8.57-13.01) | 4.96 (3.62-6.64) | 0 (0-0.48) | 18.61 (15.82-21.74) | 2.39 (1.5-3.62) | 2.86 (1.87-4.19) | 0.11 (0-0.6) | 0 (0-0.48) |
| 2018 | Male | 07-12 | 9.71 (7.79-11.97) | 5.01 (3.68-6.66) | 0 (0-0.46) | 15.32 (12.84-18.15) | 1.47 (0.8-2.47) | 4.04 (2.86-5.55) | 0.1 (0-0.58) | 0 (0-0.39) |
| 2019 | Male | 07-12 | 10.96 (8.95-13.29) | 5.55 (4.17-7.24) | 0 (0-0.49) | 15.59 (13.12-18.38) | 0.91 (0.42-1.73) | 2.88 (1.91-4.16) | 0.5 (0.16-1.18) | 0 (0-0.41) |
| 2020 | Male | 07-12 | 8.99 (7.31-10.93) | 4.62 (3.46-6.05) | 0 (0-0.48) | 15.98 (13.68-18.56) | 1.12 (0.6-1.91) | 3.32 (2.35-4.56) | 0.09 (0-0.48) | 0 (0-0.32) |
| 2021 | Male | 07-12 | 11.64 (9.89-13.60) | 3.59 (2.66-4.73) | 0.07 (0-0.39) | 14.82 (12.81-17.05) | 0.99 (0.54-1.67) | 2.02 (1.34-2.91) | 0.07 (0-0.4) | 0.14 (0.02-0.51) |
| 2022 | Male | 07-12 | 10.79 (9.12-12.67) | 6.4 (5.15-7.87) | 0 (0-0.26) | 14.47 (12.5-16.67) | 1.12 (0.64-1.82) | 2.42 (1.68-3.39) | 0 (0-0.26) | 0 (0-0.26) |
| 2016 | Male | 13-17 | 4.93 (3.42-6.89) | 6.84 (5.04-9.07) | 0.14 (0-0.78) | 8.37 (6.34-10.85) | 4.36 (2.96-6.19) | 2.55 (1.51-4.03) | 4.64 (3.19-6.51) | 0.51 (0.1-1.48) |
| 2017 | Male | 13-17 | 4.42 (3.06-6.17) | 6.5 (4.84-8.54) | 0.15 (0-0.83) | 6.73 (5.01-8.84) | 3.51 (2.33-5.08) | 3.16 (2.04-4.66) | 4 (2.74-5.65) | 0.45 (0.09-1.32) |
| 2018 | Male | 13-17 | 4.44 (3.09-6.18) | 8.45 (6.56-10.71) | 0 (0-0.53) | 8.53 (6.6-10.86) | 5.74 (4.22-7.63) | 3.19 (2.08-4.68) | 2.43 (1.48-3.75) | 0.36 (0.08-1.06) |
| 2019 | Male | 13-17 | 3.72 (2.51-5.32) | 6.31 (4.71-8.27) | 0 (0-0.58) | 6.61 (4.94-8.67) | 7.17 (5.47-9.22) | 2.39 (1.46-3.69) | 3.42 (2.29-4.91) | 0.77 (0.28-1.68) |
| 2020 | Male | 13-17 | 4.12 (2.92-5.66) | 6.92 (5.34-8.82) | 0.47 (0.1-1.36) | 6.01 (4.51-7.84) | 5.97 (4.52-7.74) | 2.73 (1.78-3.99) | 3.62 (2.52-5.04) | 0.52 (0.17-1.21) |
| 2021 | Male | 13-17 | 5.41 (4.12-6.98) | 5.67 (4.36-7.26) | 0.35 (0.1-0.9) | 6.48 (5.04-8.19) | 7.01 (5.55-8.74) | 2.75 (1.87-3.91) | 3.6 (2.58-4.89) | 0.7 (0.3-1.38) |
| 2022 | Male | 13-17 | 4.50 (3.34-5.93) | 6.61 (5.2-8.29) | 0.09 (0-0.48) | 5.90 (4.54-7.53) | 5.13 (3.9-6.61) | 2.87 (1.97-4.03) | 3.18 (2.24-4.39) | 0.69 (0.3-1.36) |
| 2016 | Male | 18-24 | 1.96 (1.18-3.05) | 13.46 (11.23-16.01) | 0.1 (0-0.56) | 2.38 (1.51-3.57) | 7.25 (5.65-9.15) | 4.73 (3.47-6.31) | 5.89 (4.46-7.63) | 0.75 (0.27-1.62) |
| 2017 | Male | 18-24 | 1.88 (1.17-2.88) | 9.73 (7.97-11.77) | 0.21 (0.03-0.76) | 2.98 (2.05-4.18) | 9.4 (7.68-11.39) | 3.67 (2.64-4.98) | 5.49 (4.2-7.05) | 0.11 (0-0.61) |
| 2018 | Male | 18-24 | 2.87 (1.98-4.04) | 9.81 (8.06-11.82) | 0 (0-0.38) | 3.25 (2.29-4.48) | 10.9 (9.07-12.99) | 5.65 (4.36-7.2) | 5.94 (4.61-7.53) | 0.61 (0.25-1.26) |
| 2019 | Male | 18-24 | 2.82 (1.94-3.95) | 13.12 (11.11-15.4) | 0 (0-0.41) | 2.93 (2.03-4.09) | 10.85 (9.04-12.91) | 5.36 (4.12-6.86) | 5.29 (4.06-6.78) | 0.56 (0.2-1.21) |
| 2020 | Male | 18-24 | 3.35 (2.43-4.50) | 11.36 (9.59-13.36) | 0 (0-0.41) | 2.61 (1.81-3.65) | 9.2 (7.62-11) | 5.23 (4.07-6.62) | 4.78 (3.67-6.12) | 0.53 (0.21-1.09) |
| 2021 | Male | 18-24 | 3.89 (2.97-5.01) | 10.63 (9.05-12.4) | 0.06 (0-0.35) | 2.42 (1.71-3.34) | 11.04 (9.44-12.84) | 4.97 (3.92-6.21) | 5.94 (4.79-7.29) | 0.52 (0.22-1.02) |
| 2022 | Male | 18-24 | 3.65 (2.76-4.73) | 11.4 (9.77-13.21) | 0 (0-0.23) | 2.00 (1.36-2.84) | 10.59 (9.03-12.34) | 5.14 (4.08-6.39) | 5.53 (4.43-6.82) | 0.57 (0.26-1.09) |

| Females: Incidence per Problem (cases/1000 person years with 95% confidence intervals) Categorized by year and age category (years) eTable 3 Females: incidence of mental health problems | | | | | | | | | | | |
| --- | --- | --- | --- | --- | --- | --- | --- | --- | --- | --- | --- |
| **year** | **sex** | **age group** | **ADHD** | **Anxiety problems** | **Eating disorders** | **Behavioral problems** | **Depressive problems** | **Sleeping problems** | **Substance abuse** | **Suicidality** |  |
| 2016 | Female | 00-06 | 1.24 (0.62-2.22) | 2.83 (1.83-4.17) | 0 (0-0.41) | 6.39 (4.83-8.30) | 0 (0-0.42) | 3.18 (2.12-4.60) | 0.45 (0.12-1.15) | 0 (0-0.5) |  |
| 2017 | Female | 00-06 | 0.5 (0.16-1.18) | 1.82 (1.08-2.88) | 0 (0-0.45) | 6.53 (5.03-8.34) | 0 (0-0.37) | 3.87 (2.74-5.31) | 0.20 (0.02-0.73) | 0 (0-0.45) |  |
| 2018 | Female | 00-06 | 1.19 (0.62-2.08) | 1.99 (1.22-3.08) | 0 (0-0.44) | 6.24 (4.78-8.00) | 0.1 (0-0.55) | 5.53 (4.16-7.19) | 0.20 (0.02-0.72) | 0 (0-0.37) |  |
| 2019 | Female | 00-06 | 1.46 (0.82-2.41) | 1.96 (1.19-3.02) | 0 (0-0.48) | 6.42 (4.95-8.18) | 0 (0-0.36) | 4.83 (3.58-6.39) | 0.19 (0.02-0.7) | 0 (0-0.39) |  |
| 2020 | Female | 00-06 | 0.6 (0.24-1.23) | 1.36 (0.78-2.22) | 0 (0-0.47) | 6.18 (4.84-7.79) | 0.17 (0.02-0.61) | 4.04 (2.97-5.37) | 0.51 (0.19-1.11) | 0 (0-0.31) |  |
| 2021 | Female | 00-06 | 1 (0.54-1.67) | 2.21 (1.5-3.14) | 0 (0-0.26) | 6.4 (5.14-7.87) | 0 (0-0.26) | 4.1 (3.1-5.31) | 0.36 (0.12-0.83) | 0 (0-0.26) |  |
| 2022 | Female | 00-06 | 1.62 (1.03-2.43) | 2.69 (1.9-3.69) | 0 (0-0.26) | 5.63 (4.45-7.01) | 0.07 (0-0.39) | 3.56 (2.64-4.69) | 0.21 (0.04-0.62) | 0 (0-0.26) |  |
| 2016 | Female | 07-12 | 4.33 (3-6.06) | 7.65 (5.83-9.84) | 0.38 (0.08-1.1) | 8.5 (6.56-10.83) | 1.63 (0.87-2.79) | 4.69 (3.3-6.46) | 0.12 (0-0.7) | 0 (0-0.56) |  |
| 2017 | Female | 07-12 | 5.27 (3.86-7.03) | 8.03 (6.26-10.15) | 0.27 (0.03-0.97) | 8.59 (6.73-10.8) | 1.8 (1.03-2.93) | 2.96 (1.94-4.34) | 0.22 (0.03-0.81) | 0.27 (0.03-0.98) |  |
| 2018 | Female | 07-12 | 4.64 (3.34-6.27) | 4.77 (3.45-6.42) | 0.13 (0-0.72) | 10.67 (8.63-13.06) | 1.41 (0.75-2.41) | 4.52 (3.24-6.13) | 0.11 (0-0.6) | 0.11 (0-0.6) |  |
| 2019 | Female | 07-12 | 5.85 (4.4-7.61) | 7.25 (5.63-9.19) | 0 (0-0.51) | 9.32 (7.44-11.52) | 1.88 (1.11-2.97) | 2.66 (1.72-3.92) | 0 (0-0.38) | 0 (0-0.42) |  |
| 2020 | Female | 07-12 | 5.07 (3.83-6.58) | 7.54 (6-9.35) | 0.13 (0-0.75) | 11.08 (9.18-13.26) | 1.96 (1.23-2.97) | 3.53 (2.51-4.83) | 0 (0-0.33) | 0.27 (0.06-0.78) |  |
| 2021 | Female | 07-12 | 4.73 (3.64-6.06) | 7.55 (6.14-9.18) | 0.07 (0-0.41) | 8.95 (7.4-10.73) | 1.93 (1.26-2.83) | 2.48 (1.71-3.49) | 0.07 (0-0.41) | 0.15 (0.02-0.53) |  |
| 2022 | Female | 07-12 | 6.37 (5.1-7.87) | 7.22 (5.85-8.8) | 0.07 (0-0.41) | 9.28 (7.71-11.08) | 2.41 (1.66-3.39) | 3.64 (2.69-4.81) | 0.07 (0-0.41) | 0.15 (0.02-0.53) |  |
| 2016 | Female | 13-17 | 4.21 (2.82-6.05) | 15.12 (12.33-18.36) | 1 (0.4-2.06) | 6.95 (5.11-9.25) | 11.06 (8.71-13.84) | 4.77 (3.28-6.7) | 3.72 (2.43-5.45) | 1.21 (0.49-2.49) |  |
| 2017 | Female | 13-17 | 3.21 (2.08-4.74) | 14.63 (12.05-17.61) | 1.21 (0.52-2.38) | 7.85 (5.99-10.1) | 15.05 (12.45-18.04) | 3.57 (2.37-5.16) | 2.02 (1.15-3.28) | 0.61 (0.17-1.56) |  |
| 2018 | Female | 13-17 | 3.27 (2.14-4.79) | 11.8 (9.51-14.47) | 1.92 (1.02-3.29) | 5.28 (3.79-7.17) | 15.45 (12.83-18.45) | 4.5 (3.15-6.23) | 2.59 (1.61-3.97) | 2.47 (1.51-3.82) |  |
| 2019 | Female | 13-17 | 3.49 (2.32-5.05) | 17.61 (14.8-20.81) | 2.27 (1.24-3.81) | 7.05 (5.31-9.17) | 16.49 (13.79-19.57) | 5.7 (4.18-7.61) | 2.56 (1.59-3.92) | 1.06 (0.46-2.1) |  |
| 2020 | Female | 13-17 | 4.17 (2.95-5.73) | 16.07 (13.54-18.93) | 1.62 (0.78-2.98) | 6.43 (4.87-8.33) | 14.59 (12.21-17.3) | 4.05 (2.85-5.58) | 2.05 (1.23-3.19) | 1.94 (1.15-3.06) |  |
| 2021 | Female | 13-17 | 6.16 (4.76-7.83) | 18.64 (16.12-21.45) | 2.56 (1.7-3.7) | 6.97 (5.46-8.76) | 20.92 (18.26-23.86) | 4.08 (2.97-5.48) | 2.1 (1.33-3.15) | 1.74 (1.05-2.71) |  |
| 2022 | Female | 13-17 | 7.69 (6.13-9.53) | 20.01 (17.4-22.91) | 1.36 (0.76-2.24) | 7.68 (6.1-9.55) | 16 (13.69-18.58) | 2.66 (1.78-3.82) | 2.53 (1.68-3.66) | 2.17 (1.39-3.23) |  |
| 2016 | Female | 18-24 | 2.64 (1.71-3.89) | 24.77 (21.57-28.3) | 1.68 (0.96-2.73) | 2.13 (1.3-3.29) | 17.79 (15.15-20.75) | 6.31 (4.81-8.14) | 2.22 (1.37-3.39) | 0.89 (0.36-1.83) |  |
| 2017 | Female | 18-24 | 2.23 (1.43-3.32) | 25.17 (22.14-28.5) | 0.56 (0.18-1.3) | 2.44 (1.6-3.58) | 16.88 (14.46-19.58) | 8.02 (6.41-9.92) | 2.32 (1.5-3.43) | 1.23 (0.62-2.21) |  |
| 2018 | Female | 18-24 | 2 (1.25-3.02) | 27.25 (24.12-30.68) | 0.97 (0.44-1.84) | 2.11 (1.34-3.17) | 19.61 (17.02-22.48) | 6.9 (5.43-8.65) | 2.9 (1.98-4.09) | 1.36 (0.76-2.24) |  |
| 2019 | Female | 18-24 | 3.59 (2.58-4.87) | 27.2 (24.12-30.57) | 1.5 (0.8-2.56) | 1.77 (1.08-2.74) | 18.51 (16.04-21.26) | 6.66 (5.24-8.35) | 1.92 (1.2-2.9) | 1.14 (0.59-1.99) |  |
| 2020 | Female | 18-24 | 3.02 (2.15-4.12) | 23.01 (20.36-25.9) | 1.49 (0.8-2.55) | 1.09 (0.6-1.83) | 15.21 (13.11-17.56) | 6.8 (5.45-8.39) | 3.07 (2.19-4.18) | 0.92 (0.48-1.61) |  |
| 2021 | Female | 18-24 | 5.57 (4.45-6.89) | 27.77 (25.09-30.65) | 1.49 (0.94-2.23) | 1.91 (1.28-2.75) | 22.07 (19.73-24.63) | 7.54 (6.22-9.05) | 2.6 (1.86-3.54) | 0.72 (0.36-1.28) |  |
| 2022 | Female | 18-24 | 6.41 (5.21-7.80) | 28.72 (26.00-31.64) | 0.83 (0.44-1.41) | 1.04 (0.60-1.69) | 18.75 (16.60-21.11) | 5.59 (4.47-6.91) | 3.39 (2.54-4.44) | 1.35 (0.83-2.06) |  |

## eTable 4 Monthly trends of incidence

| Monthly trends in incidence per problem category | | | | | | | | | | | | | | | | | |
| --- | --- | --- | --- | --- | --- | --- | --- | --- | --- | --- | --- | --- | --- | --- | --- | --- | --- |
|  |  | ADHD | | Anxiety problems | | Eating disorders | | Behavioural problems | | Depressive problems | | Sleeping problems | | Substance abuse | | Suicidality | |
|  |  | Trend (95%CI) | P value | Trend (95%CI) | P value | Trend (95%CI) | P value | Trend (95%CI) | P value | Trend (95%CI) | P value | Trend (95%CI) | P value | Trend (95%CI) | P value | Trend (95%CI) | P value |
| Overall |  | 1.004 (1.002-1.006) | <0.001 | 1.001 (1-1.002) | 0.164 | 1.000 (0.994-1.005)* | 0.945 | 0.999 (0.997-1.000) | 0.106 | 1.003 (1.001-1.005) | 0.004 | 0.999 (0.997-1.000)* | 0.095 | 0.999 (0.997-1.001)* | 0.400 | 1.004 (0.999-1.009)* | 0.122 |
| Male overall |  | 1.001 (0.999-1.003)* | 0.363 | 0.999 (0.997-1.001) | 0.221 | Too low number | 0.363 | 0.998 (0.996-1.000) | 0.026 | 1.003 (1.000-1.005) | 0.04 | 1.000 (0.998-1.002)* | 0.953 | 0.998 (0.995-1.001)* | 0.192 | 1.004 (0.994-1.013)* | 0.434 |
| Female overall |  | 1.01 (1.007-1.013) | <0.001 | 1.002 (1.001-1.004) | 0.003 | 1.001 (0.995-1.006)* | 0.824 | 0.999 (0.997-1.002) | 0.645 | 1.003 (1.001-1.005) | 0.01 | 0.998 (0.995-1.000)* | 0.028 | 1.001 (0.997-1.005)* | 0.740 | 1.004 (0.998-1.01)* | 0.179 |
| Male | 0-6 | 0.994 (0.989-0.999) | 0.016 | 0.992 (0.984-1.000) | 0.061 | Too low number** |  | 0.999 (0.996-1.002) | 0.494 | Too low number** |  | 1.001 (0.997-1.005)* | 0.637 | Too low number** |  | No cases |  |
|  | 7-12 | 1.000 (0.997-1.003) | 0.925 | 1.001 (0.996-1.005)* | 0.797 | Too low number** |  | 0.998 (0.995-1.000) | 0.062 | 0.993 (0.985-1.001)* | 0.083 | 0.995 (0.989-1.000)* | 0.068 | Too low number** |  | Too low number** |  |
|  | 13-17 | 1.001 (0.996-1.006)* | 0.790 | 0.998 (0.994-1.002)* | 0.365 | Too low number** |  | 0.996 (0.991-1.000) | 0.047 | 1.005 (1.000-1.009)* | 0.044 | 1.000 (0.994-1.006)* | 0.906 | 0.997 (0.992-1.003) | 0.295 | 1.006 (0.991-1.02)* | 0.437 |
|  | 18-24 | 1.010 (1.004-1.015)* | <0.001 | 0.999 (0.997-1.002) | 0.696 | Too low number** |  | 0.996 (0.991-1.002)* | 0.158 | 1.003 (1.000-1.006) | 0.037 | 1.002 (0.998-1.006)* | 0.364 | 0.999 (0.996-1.003)* | 0.783 | 1.001 (0.989-1.014)* | 0.823 |
| Female | 0-6 | 1.005 (0.996-1.014)* | 0.275 | 1.001 (0.994-1.007)* | 0.817 | No cases |  | 0.999 (0.995-1.002)* | 0.469 | Too low number** |  | 0.999 (0.995-1.004)* | 0.770 | Too low number** |  | No cases |  |
|  | 7-12 | 1.003 (0.999-1.007)* | 0.147 | 1.000 (0.997-1.004) | 0.811 | Too low number** |  | 1.001 (0.998-1.004)* | 0.672 | 1.005 (0.998-1.012)* | 0.141 | 0.996 (0.991-1.001)* | 0.130 | Too low number** |  | Too low number** |  |
|  | 13-17 | 1.012 (1.007-1.018)* | <0.001 | 1.005 (1.002-1.008) | 0.001 | 1.005 (0.997-1.014) | 0.218 | 1.001 (0.997-1.005) | 0.660 | 1.005 (1.002-1.008) | 0.002 | 0.995 (0.99-1.000)* | 0.074 | 0.996 (0.989-1.003)* | 0.227 | 1.008 (1.000-1.017)* | 0.056 |
|  | 18-24 | 1.017 (1.011-1.022) | <0.001 | 1.002 (1.000-1.004) | 0.139 | 0.999 (0.991-1.007)* | 0.822 | 0.991 (0.984-0.998)* | 0.007 | 1.002 (0.999-1.004) | 0.165 | 0.998 (0.995-1.002)* | 0.321 | 1.005 (1-1.01)* | 0.075 | 1.000 (0.991-1.009)* | 0.924 |

Monthly trends in relative rate with 95% confidence interval.

* there was no over dispersion (i.e. a Poisson distribution was used)

** unstable model due to low number of cases

| Monthly trends in Contact rates per problem category | | | | | | | | | | | | | | | | | |
| --- | --- | --- | --- | --- | --- | --- | --- | --- | --- | --- | --- | --- | --- | --- | --- | --- | --- |
|  |  | ADHD | | Anxiety problems | | Eating disorders | | Behavioral problems | | Depressive problems | | Sleeping problems | | Substance abuse | | Suicidality | |
|  |  | Trend (95%CI) | P value | Trend (95%CI) | P value | Trend (95%CI) | P value | Trend (95%CI) | P value | Trend (95%CI) | P value | Trend (95%CI) | P value | Trend (95%CI) | P value | Trend (95%CI) | P value |
| Overall |  | 1.005 (1.004-1.006) | <0.001 | 1.008 (1.007-1.009) | <0.001 | 1.007 (1.005-1.010) | <0.001 | 1.005 (1.004-1.006) | <0.001 | 1.008 (1.007-1.009) | <0.001 | 1.002 (1.001-1.004) | 0.001 | 1.002 (1.001-1.004) | 0.007 | 1.013 (1.009-1.016) | <0.001 |
| Male overall |  | 1.003 (1.002-1.004) | <0.001 | 1.005 (1.003-1.006) | <0.001 | 1.000 (0.989-1.011) | 0.975 | 1.004 (1.003-1.005) | <0.001 | 1.008 (1.007-1.010) | <0.001 | 1.001 (0.999-1.002) | 0.407 | 1.002 (1.000-1.004) | 0.014 | 1.010 (1.003-1.018) | 0.008 |
| Female overall |  | 1.008 (1.007-1.010) | <0.001 | 1.009 (1.008-1.010) | <0.001 | 1.008 (1.005-1.011) | <0.001 | 1.005 (1.004-1.007) | <0.001 | 1.007 (1.006-1.009) | <0.001 | 1.003 (1.002-1.005) | <0.001 | 1.002 (1.000-1.004) | 0.080 | 1.014 (1.010-1.017) | <0.001 |
| Male | 0-6 | 0.994 (0.990-0.997) | <0.001 | 0.999 (0.991-1.006) | 0.726 | Too low number** |  | 1.006 (1.003-1.008) | <0.001 | Too low number** |  | 1.003 (0.998-1.007) | 0.219 | Too low number** |  | No cases |  |
|  | 7-12 | 1.000 (0.998-1.001) | 0.538 | 1.005 (1.001-1.009) | 0.006 | Too low number** |  | 1.005 (1.003-1.007) | <0.001 | 0.997 (0.992-1.003) | 0.316 | 0.997 (0.993-1.002) | 0.221 | Too low number** |  | Too low number** |  |
|  | 13-17 | 1.003 (1.001-1.004) | <0.001 | 1.004 (1.002-1.006) | <0.001 | 1.038 (1.020-1.056) | <0.001 | 1.002 (1.000-1.004) | 0.022 | 1.015 (1.012-1.018) | <0.001 | 1.002 (0.998-1.007) | 0.272 | 0.996 (0.992-0.999) | 0.026 | 1.006 (0.995-1.018) | 0.262 |
|  | 18-24 | 1.009 (1.007-1.01) | <0.001 | 1.005 (1.004-1.007) | <0.001 | 0.965 (0.946-0.984) | <0.001 | 1.002 (1.000-1.004) | 0.081 | 1.007 (1.005-1.009) | <0.001 | 1.001 (0.998-1.004) | 0.497 | 1.004 (1.002-1.006) | <0.001 | 1.015 (1.004-1.025) | 0.005 |
| Female | 0-6 | 0.997 (0.990-1.004) | 0.380 | 1.008 (1.003-1.013) | 0.001 | no cases |  | 1.003 (1.000-1.006) | 0.038 | Too low number** |  | 1.003 (1.000-1.007)* | 0.054 | Too low number** |  | No cases |  |
|  | 7-12 | 1.003 (1.001-1.005) | 0.002 | 1.008 (1.006-1.011) | <0.001 | Too low number** |  | 1.008 (1.005-1.010) | <0.001 | 1.014 (1.010-1.018) | <0.001 | 1.004 (0.999-1.008) | 0.085 | Too low number** |  | Too low number** |  |
|  | 13-17 | 1.009 (1.007-1.011) | <0.001 | 1.013 (1.011-1.015) | <0.001 | 1.017 (1.012-1.022) | <0.001 | 1.006 (1.004-1.008) | <0.001 | 1.009 (1.007-1.012) | <0.001 | 0.999 (0.995-1.003) | 0.780 | 0.994 (0.988-0.999) | 0.022 | 1.012 (1.006-1.018) | <0.001 |
|  | 18-24 | 1.013 (1.011-1.015) | <0.001 | 1.008 (1.007-1.009) | <0.001 | 1.005 (1.002-1.007) | 0.001 | 1.001 (0.998-1.004) | 0.401 | 1.007 (1.005-1.008) | <0.001 | 1.005 (1.003-1.007) | <0.001 | 1.004 (1.001-1.006) | 0.003 | 1.015 (1.011-1.020) | <0.001 |

## eTable 5 Monthly trends of consultations

Monthly trends in relative rate with 95% confidence interval.

* there was no over dispersion (i.e. a Poisson distribution was used)

** unstable model due to low number of cases

| Monthly trends in Contact rates per problem category | | | | | | | | | | | | | | | | | |
| --- | --- | --- | --- | --- | --- | --- | --- | --- | --- | --- | --- | --- | --- | --- | --- | --- | --- |
|  |  | ADHD | | Anxiety problems | | Eating disorders | | Behavioral problems | | Depressive problems | | Sleeping problems | | Substance abuse | | Suicidality | |
|  |  | Trend (95%CI) | P value | Trend (95%CI) | P value | Trend (95%CI) | P value | Trend (95%CI) | P value | Trend (95%CI) | P value | Trend (95%CI) | P value | Trend (95%CI) | P value | Trend (95%CI) | P value |
| Overall |  | No autocorrelation |  | 1.008 (1.007-1.009) | <0.001 | 1.007 (1.005-1.010) | <0.001 | No autocorrelation |  | 1.008 (1.007-1.009) | <0.001 | No autocorrelation |  | 1.002 (1.001-1.004) | 0.004 | No autocorrelation |  |
| Male overall |  | No autocorrelation |  | 1.005 (1.003-1.006) | <0.001 | 0.998 (0.988-1.008) | 0.687 | No autocorrelation |  | No autocorrelation |  | No autocorrelation |  | 1.002 (1.000-1.004) | 0.012 | No autocorrelation |  |
| Female overall |  | No autocorrelation |  | 1.009 (1.009-1.010) | <0.001 | No autocorrelation |  | No autocorrelation |  | 1.007 (1.006-1.008) | <0.001 | No autocorrelation |  | 1.002 (1.000-1.004) | 0.068 | No autocorrelation |  |
| Male | 0-6 | No autocorrelation |  | No autocorrelation |  | Too low number |  | No autocorrelation |  | Too low number |  | No autocorrelation |  | Too low number |  | No cases |  |
|  | 7-12 | No autocorrelation |  | 1.005 (1.002-1.008) | 0.001 | Too low number |  | No autocorrelation |  | 0.998 (0.993-1.002) | 0.321 | No autocorrelation |  | Too low number |  | Too low number |  |
|  | 13-17 | 1.003 (1.002-1.004) | <0.001 | 1.004 (1.002-1.006) | <0.001 | 1.034 (1.017-1.052) | <0.001 | No autocorrelation |  | 1.015 (1.012-1.018) | <0.001 | 1.003 (0.998-1.007) | 0.204 | No autocorrelation |  | No autocorrelation |  |
|  | 18-24 | No autocorrelation |  | 1.005 (1.003-1.007) | <0.001 | 0.963 (0.944-0.980) | <0.001 | No autocorrelation |  | 1.007 (1.005-1.009) | <0.001 | No autocorrelation |  | 1.004 (1.002-1.006) | <0.001 | No autocorrelation |  |
| Female | 0-6 | No autocorrelation |  | No autocorrelation |  | no cases |  | No autocorrelation |  | Too low number |  | No autocorrelation |  | Too low number |  | No cases |  |
|  | 7-12 | No autocorrelation |  | 1.008 (1.006-1.010) | <0.001 | Too low number |  | No autocorrelation |  | No autocorrelation |  | No autocorrelation |  | Too low number |  | Too low number |  |
|  | 13-17 | 1.009 (1.007-1.010) | <0.001 | 1.013 (1.012-1.015) | <0.001 | 1.017 (1.012-1.023) | <0.001 | No autocorrelation |  | 1.009 (1.008-1.011) | <0.001 | 1.000 (0.996-1.004) | 0.780 | No autocorrelation |  | No autocorrelation |  |
|  | 18-24 | 1.013 (1.011-1.015) | <0.001 | No autocorrelation |  | No autocorrelation |  | 1.001 (0.998-1.003) | 0.563 | 1.006 (1.005-1.007) | <0.001 | 1.005 (1.002-1.007) | <0.001 | No autocorrelation |  | No autocorrelation |  |
|  |  |  |  |  |  |  |  |  |  |  |  |  |  |  |  |  |  |

## eTable 6 Monthly trends of consultation rates, after correction for autocorrelation

| Time trend analysis of impact of COVID-19 period on mental health problems incidence | | | | | | |
| --- | --- | --- | --- | --- | --- | --- |
|  | Trend 2016-March 2020 | | Trend 2016-2022 | | COVID-19 period March-2020 until April-2022 | |
|  | trend (95% CI) | p | trend (95% CI) | p | Expected n (95%CI) | observed |
| ADHD | 1.002 (0.998-1.005) | 0.427 | 1.004 (1.002-1.006) | <0.001 | 939 (740-1194) | 977 |
| Anxiety problems | 1.001 (0.999-1.004) | 0.407 | 1.001 (1-1.002) | 0.164 | 2068 (1763-2426) | 1968 |
| Eating disorders | 1.001 (0.988-1.015) | 0.860 | 1.000 (0.994-1.005) | 0.945 | 78 (34-183) | 86 |
| Behavioral problems | 0.998 (0.995-1.002) | 0.336 | 0.999 (0.997-1.000) | 0.106 | 1500 (1197-1879) | 1480 |
| Depressive problems | 1.006 (1.003-1.009) | <0.001 | 1.003 (1.001-1.005) | 0.004 | 1700 (1398-2067) | 1483 |
| Sleeping problems | 1.006 (1.003-1.009) | <0.001 | 0.999 (0.997-1.000) | 0.095 | 1023 (814-1286) | 878 |
| Substance abuse | 0.996 (0.99-1.001) | 0.130 | 0.999 (0.997-1.001) | 0.400 | 351 (247-498) | 401 |
| Suicidality | 1.005 (0.992-1.017) | 0.461 | 1.004 (0.999-1.009) | 0.122 | 115 (55-244) | 107 |

## eTable 7 Monthly trends of incidence, before March 2020 and for 2016-2022

## eTable 8 Monthly trends of consultation rates, before March 2020 and for 2016-2022

| Time trend analysis of impact of COVID-19 period on mental health problems consultation rates | | | | | | |
| --- | --- | --- | --- | --- | --- | --- |
|  | Trend 2016-March 2020 | | Trend 2016-2022 | | COVID-19 period March-2020 until April-2022 | |
|  | trend (95% CI) | p | trend (95% CI) | p | Expected n (95%CI) | observed |
| ADHD | 1.005 (1.003-1.007) | <0.001 | 1.005 (1.004-1.006) | <0.001 | 11712 (10439-13140) | 11367 |
| Anxiety problems | 1.007 (1.005-1.009) | <0.001 | 1.008 (1.007-1.009) | <0.001 | 13048 (11669-14589) | 13370 |
| Eating disorders | 1.009 (1.005-1.014) | <0.001 | 1.007 (1.005-1.010) | <0.001 | 1171 (880-1559) | 1207 |
| Behavioral problems | 1.003 (1.001-1.005) | <0.001 | 1.005 (1.004-1.006) | <0.001 | 8104 (7214-9105) | 8482 |
| Depressive problems | 1.010 (1.008-1.012) | <0.001 | 1.008 (1.007-1.009) | <0.001 | 14268 (12999-15661) | 13161 |
| Sleeping problems | 1.002 (0.999-1.004) | 0.289 | 1.002 (1.001-1.004) | 0.001 | 2375 (1991-2834) | 2472 |
| Substance abuse | 1.003 (1.000-1.007) | 0.086 | 1.002 (1.001-1.004) | 0.007 | 2443 (1967-3035) | 2524 |
| Suicidality | 1.019 (1.011-1.028) | <0.001 | 1.013 (1.009-1.016) | <0.001 | 770 (462-1283) | 565 |

Expected monthly incidence based on pre pandemic trend vs observed incidence. Grey area represents COVID pandemic with dark grey area first lockdown measures in the Netherlands

## eFigure 1a modelled monthly incidence rate vs observed incidence


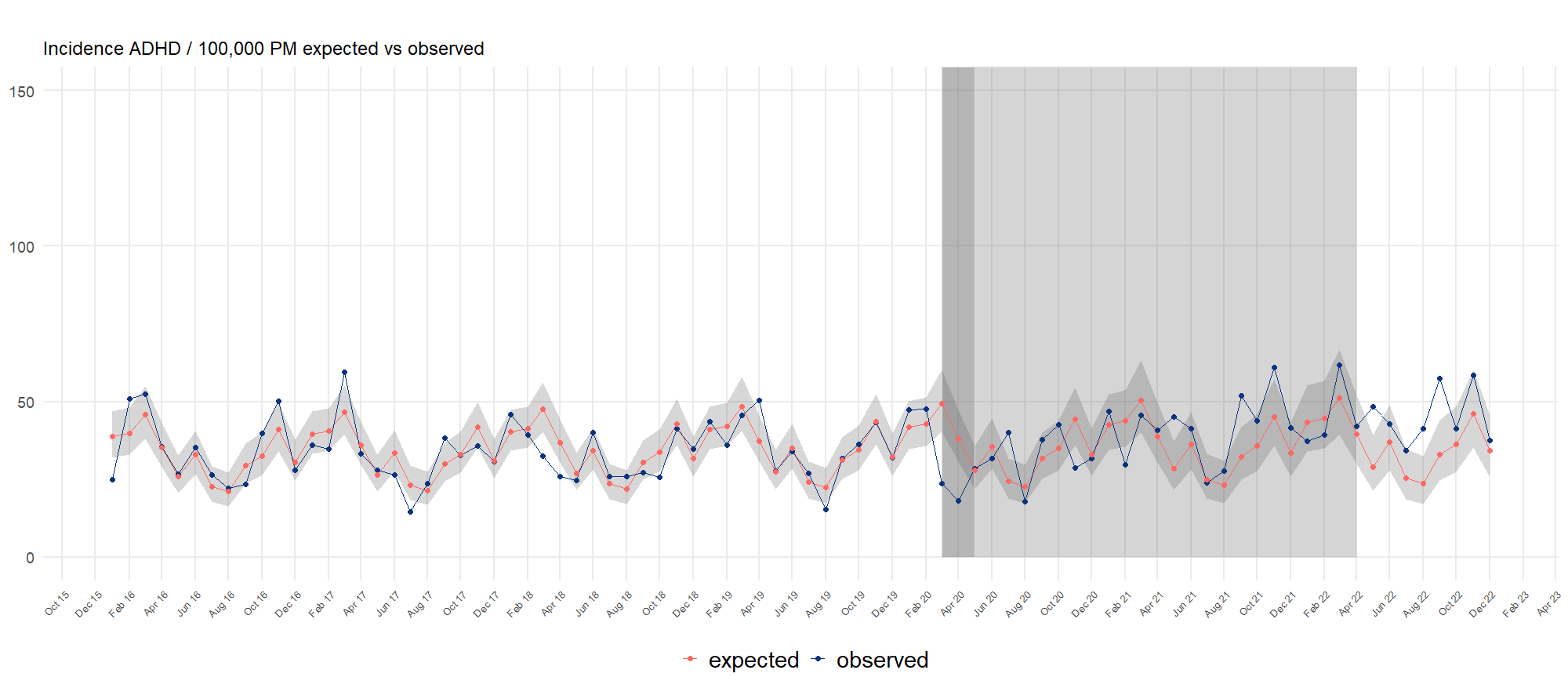


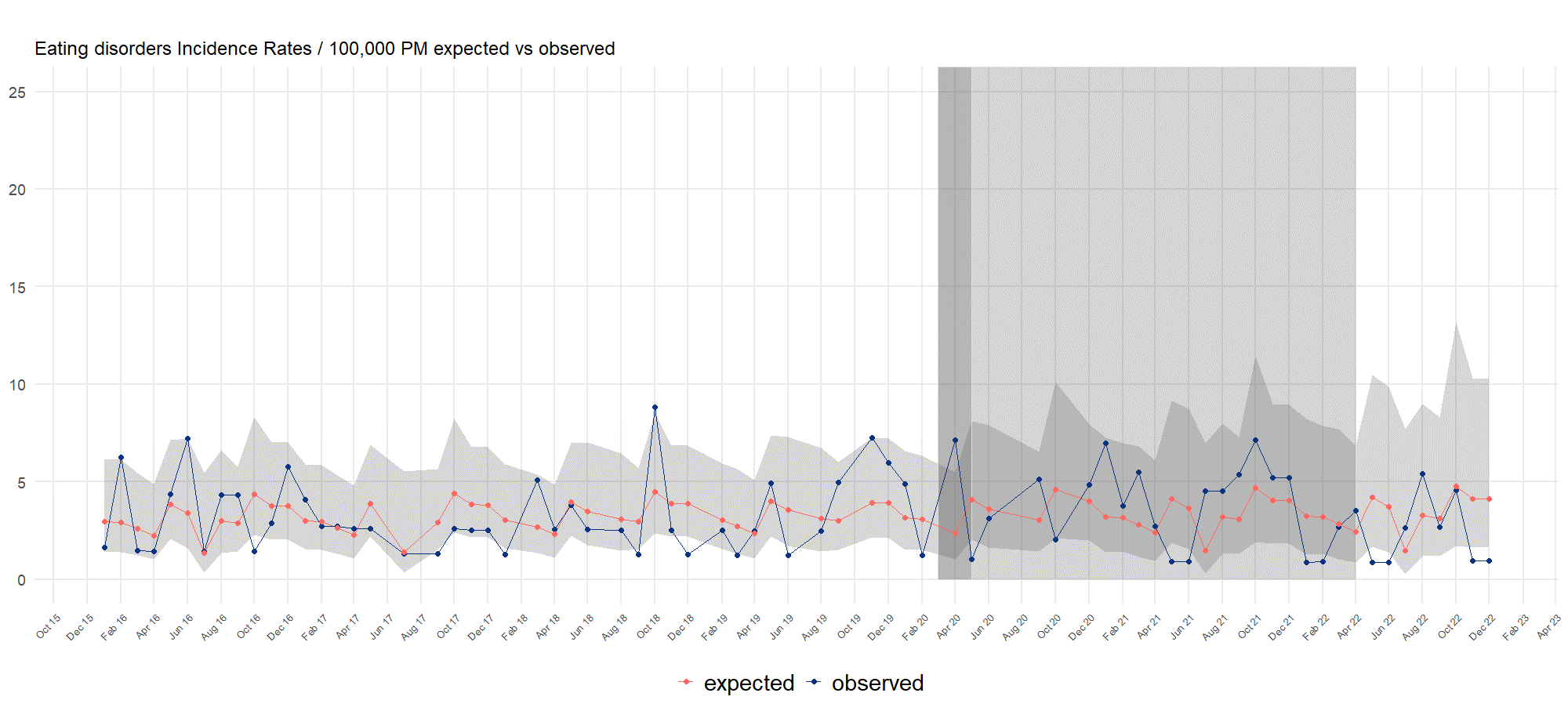

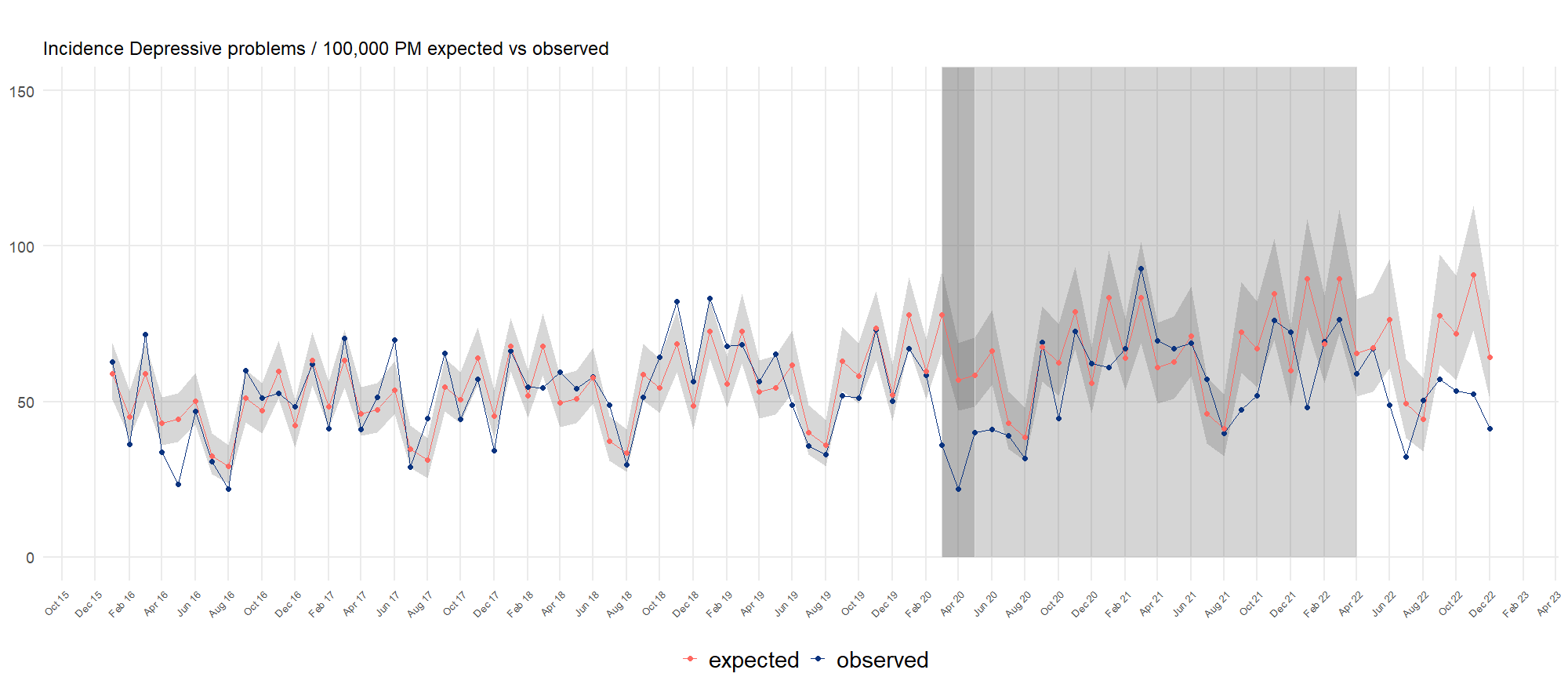

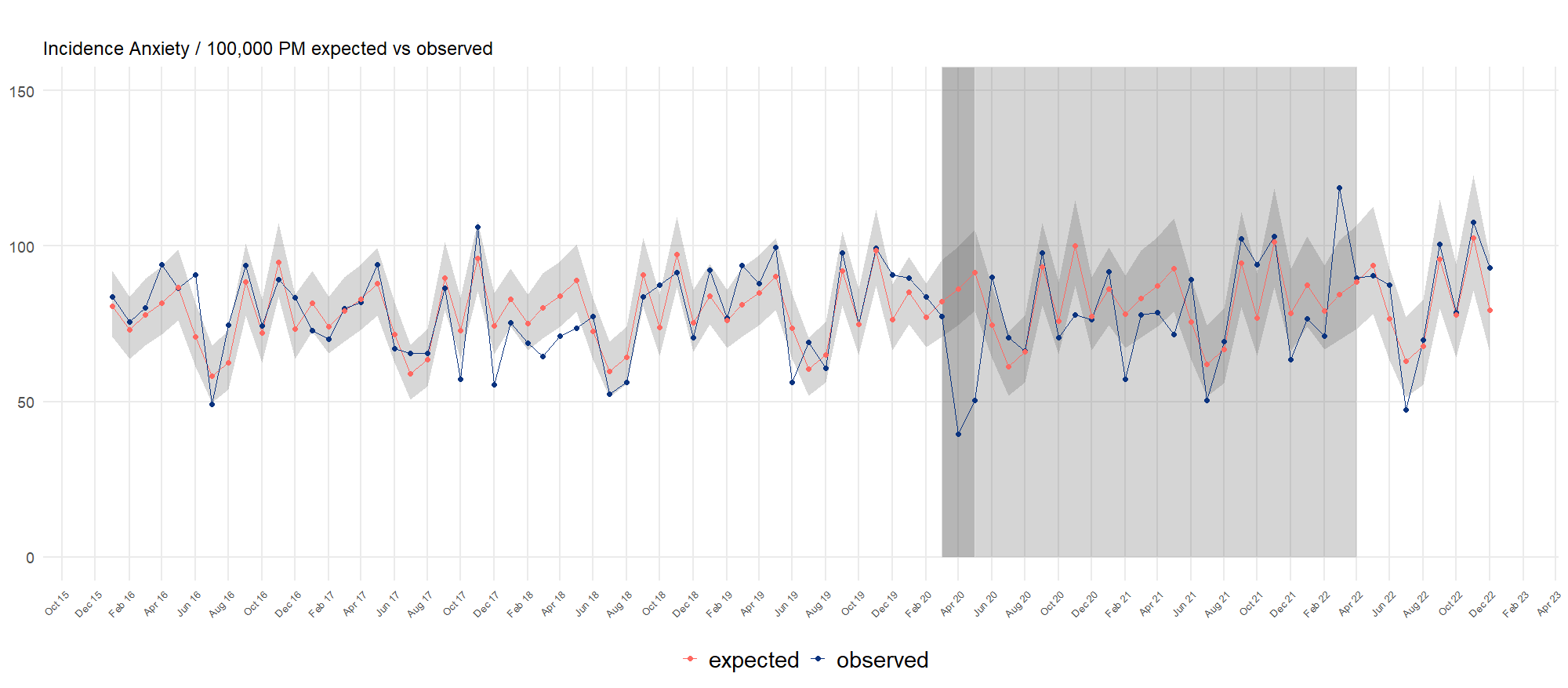

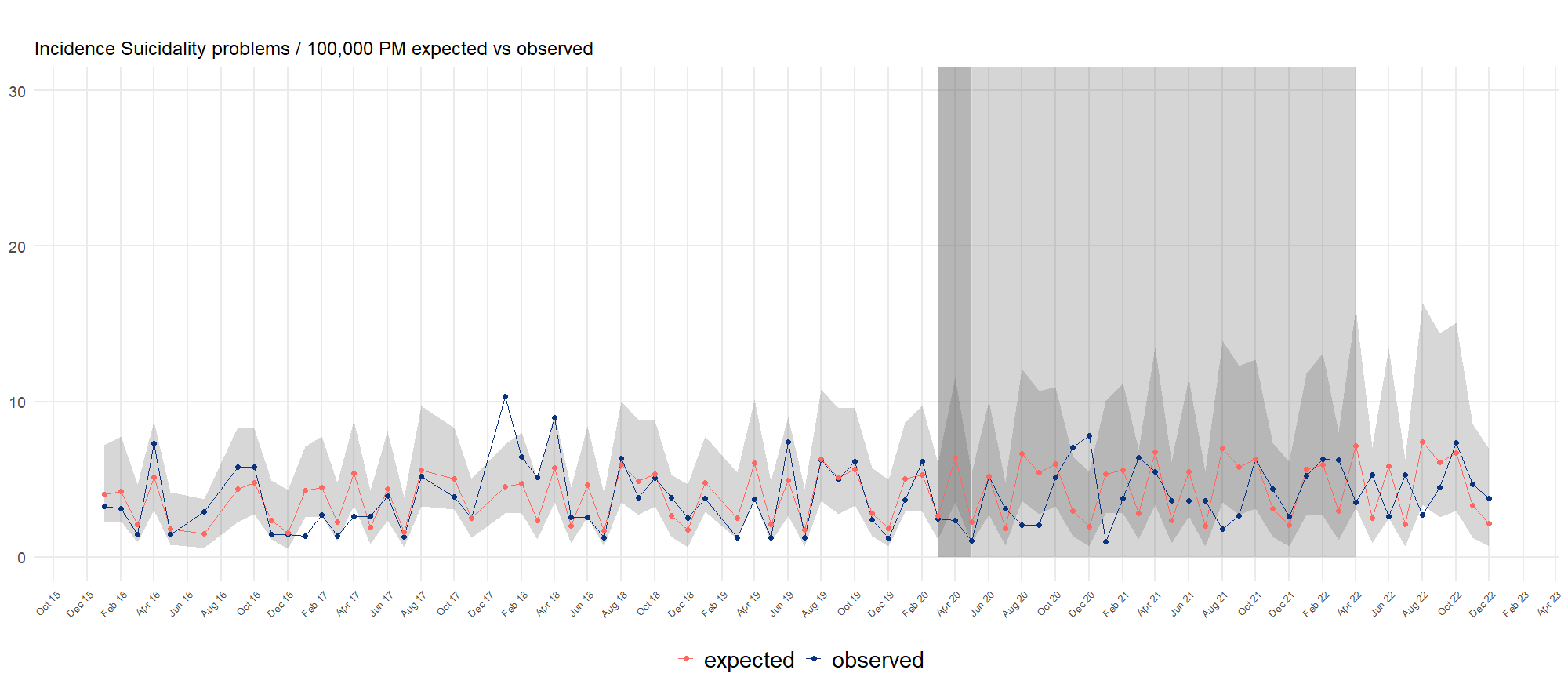

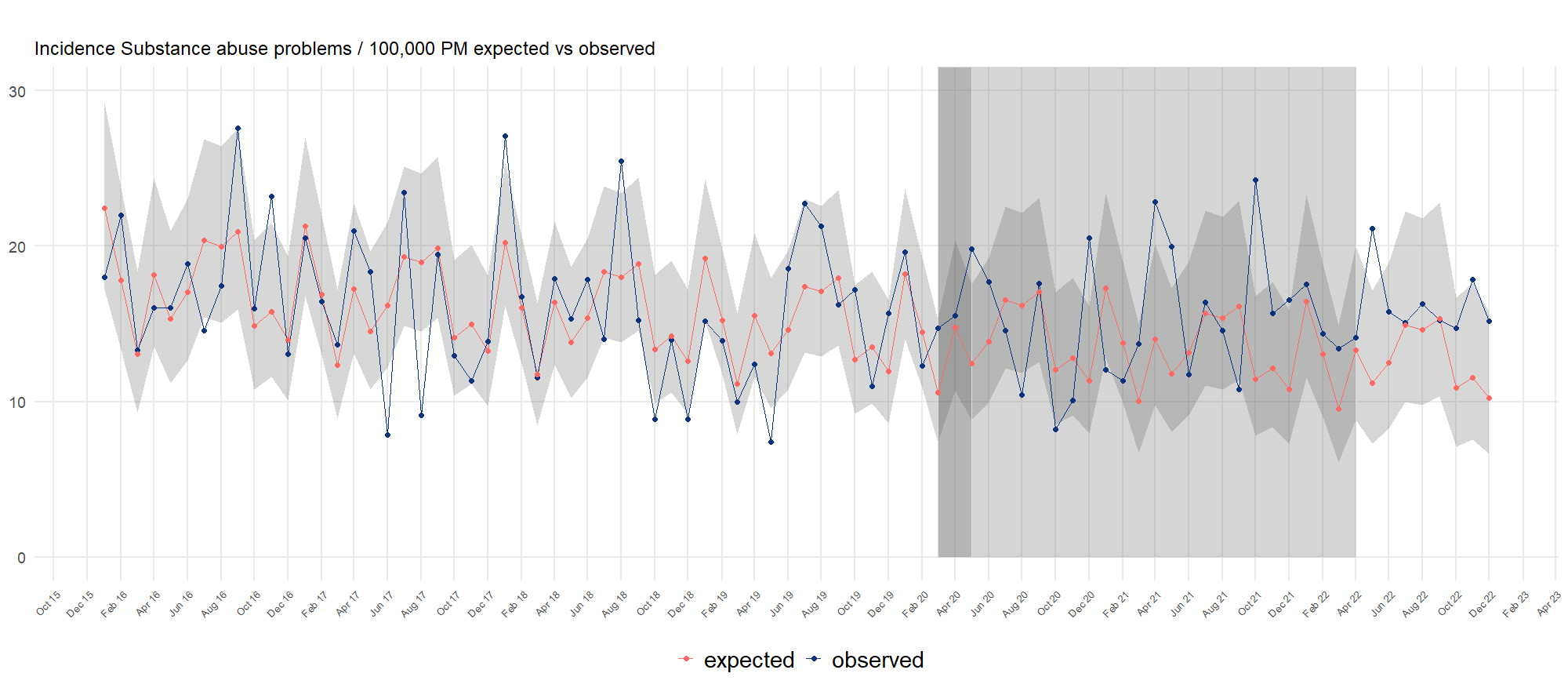

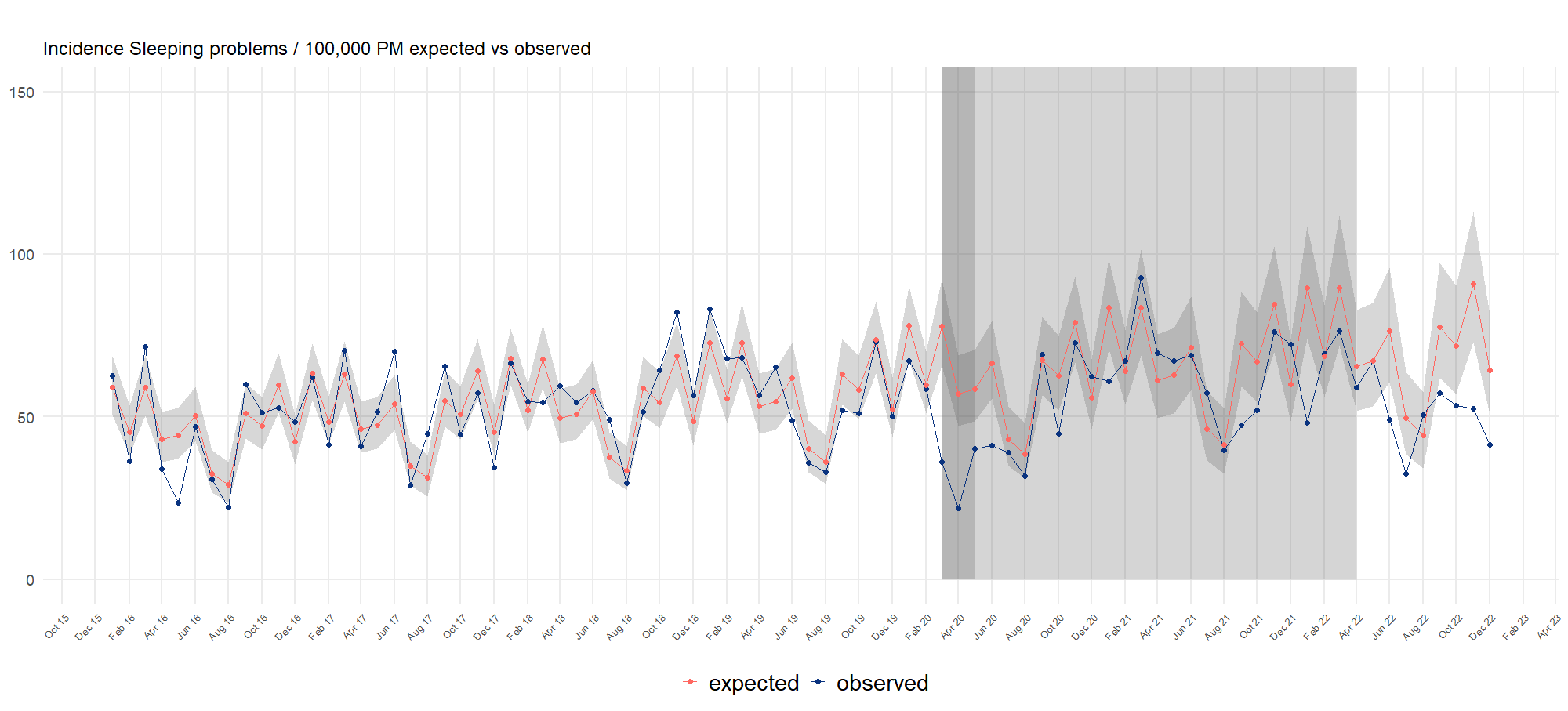

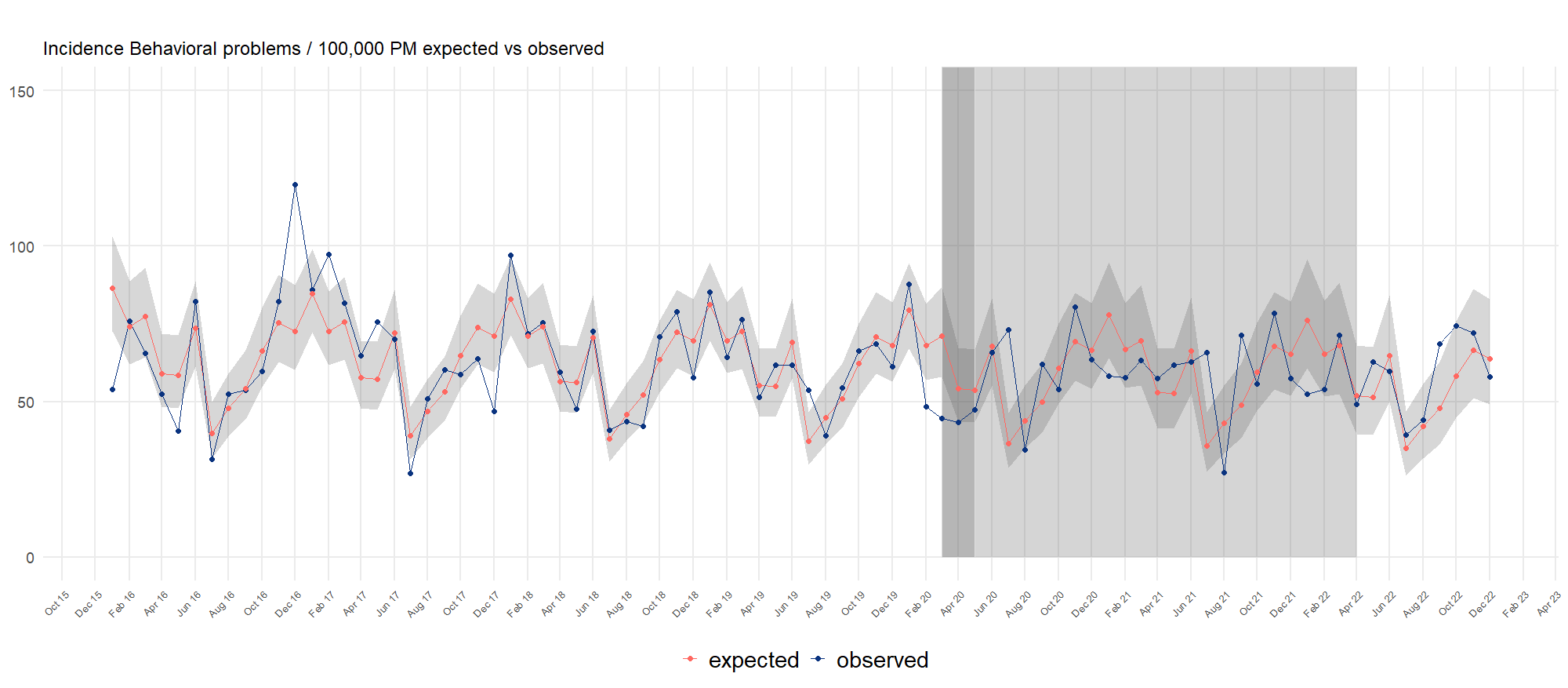


## eFigure 1a modelled monthly incidence rate vs observed incidence - continued

## eFigure 1b modelled monthly consultation rate vs observed consultation rate

Expected monthly consultations (contacts) based on pre pandemic trend vs observed consultations (contacts). Grey area represents COVID pandemic with dark grey area first lockdown measures in the Netherlands


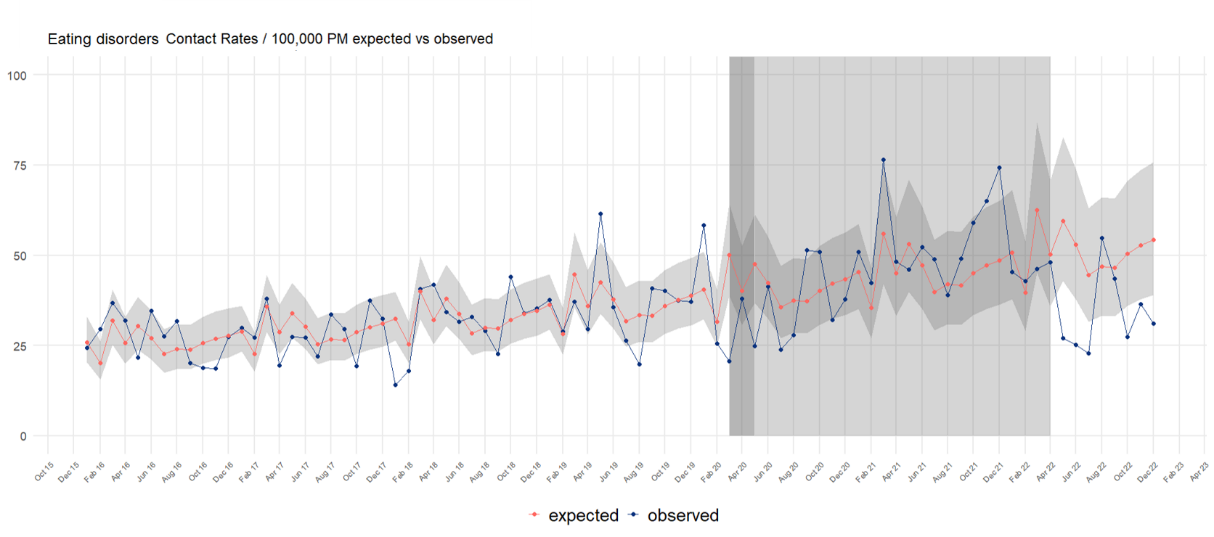

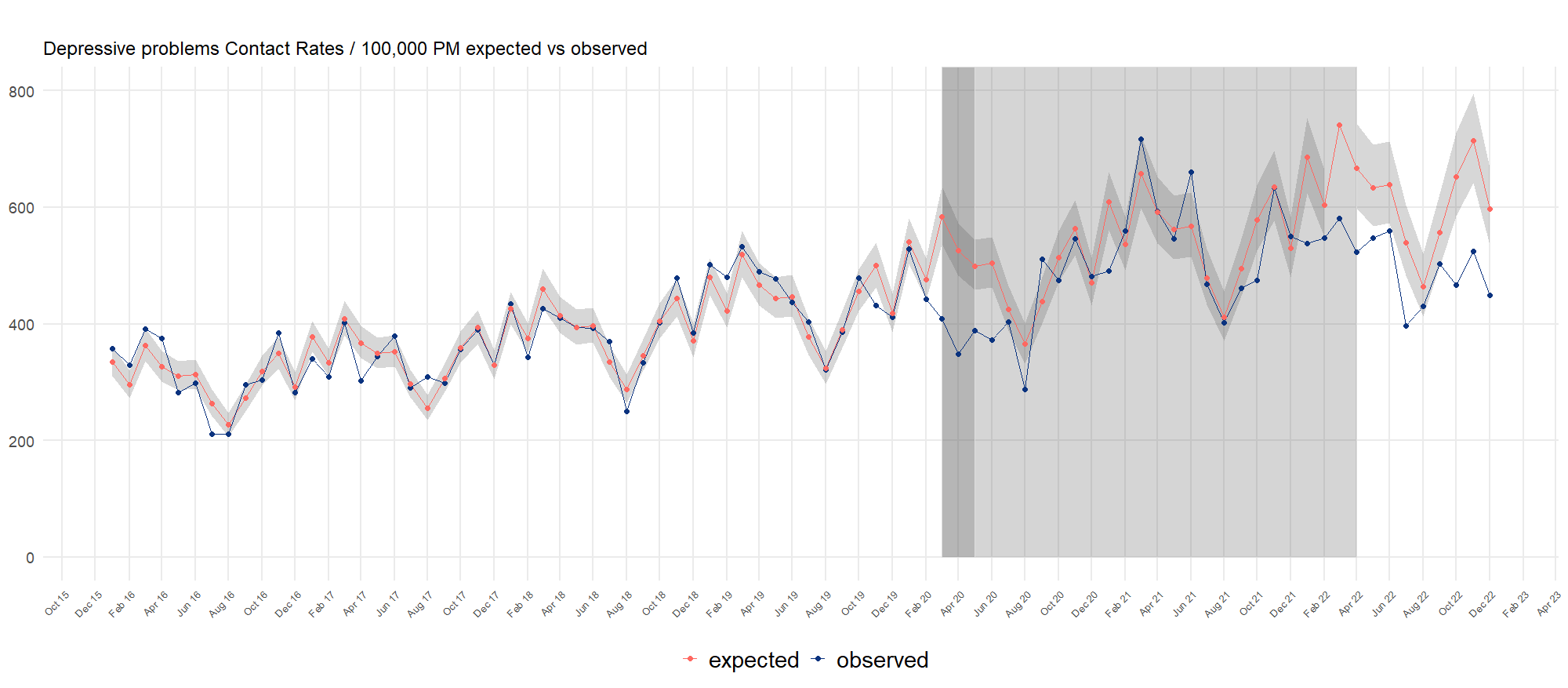

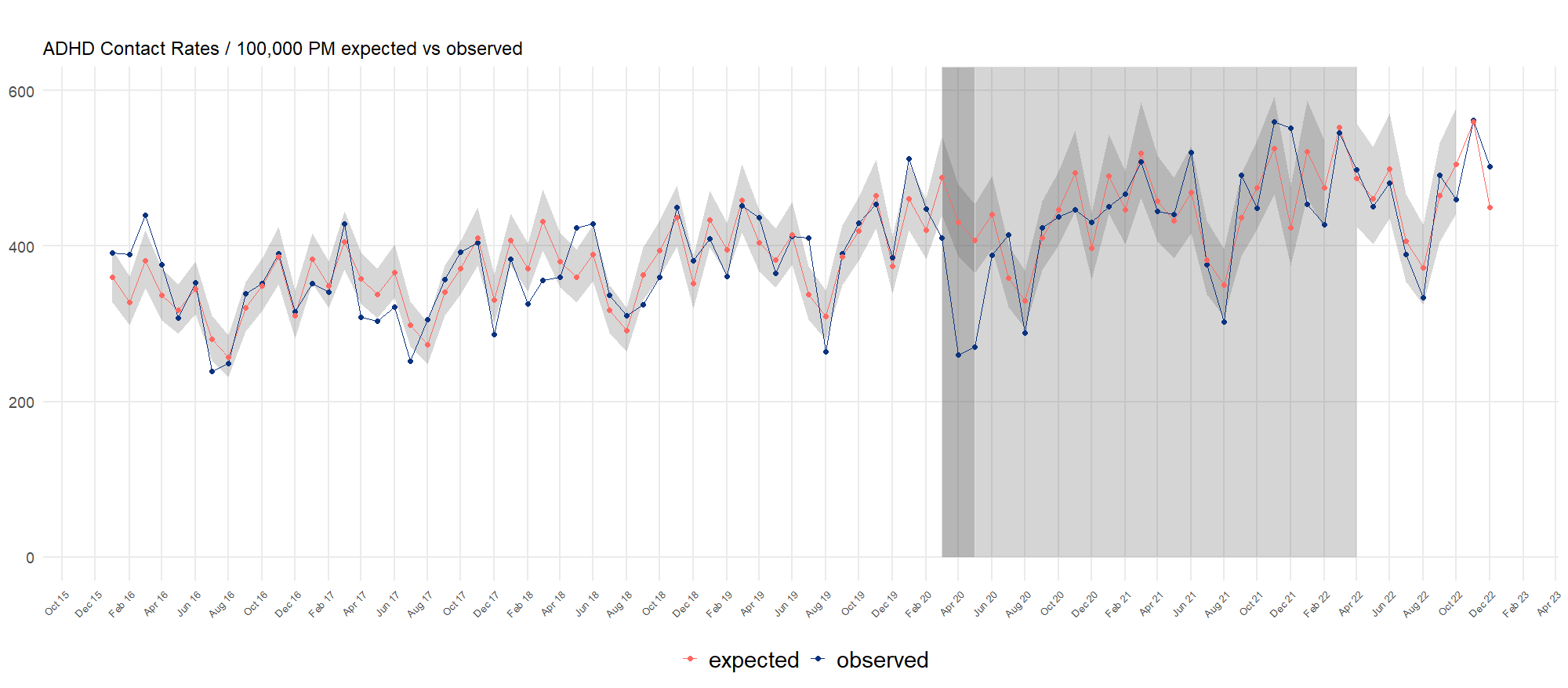

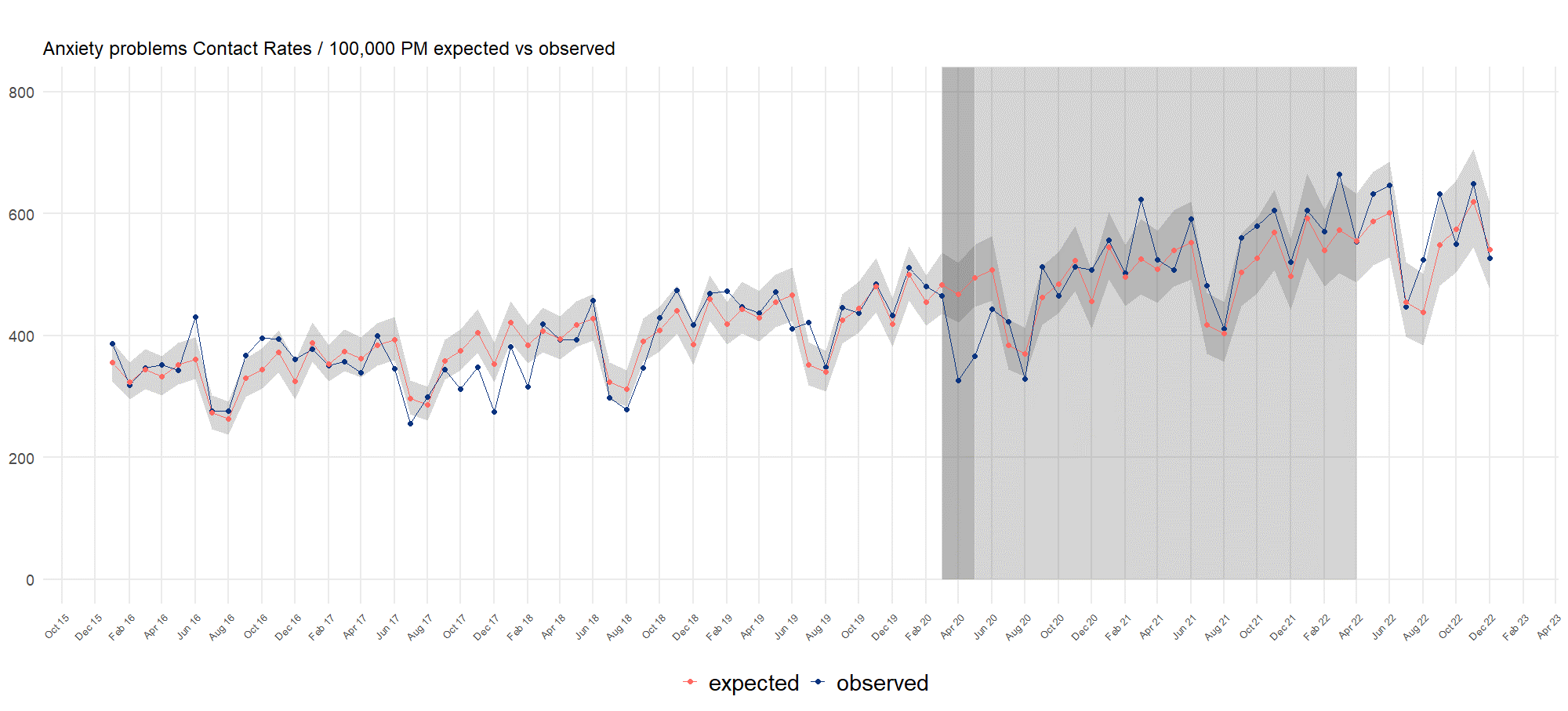


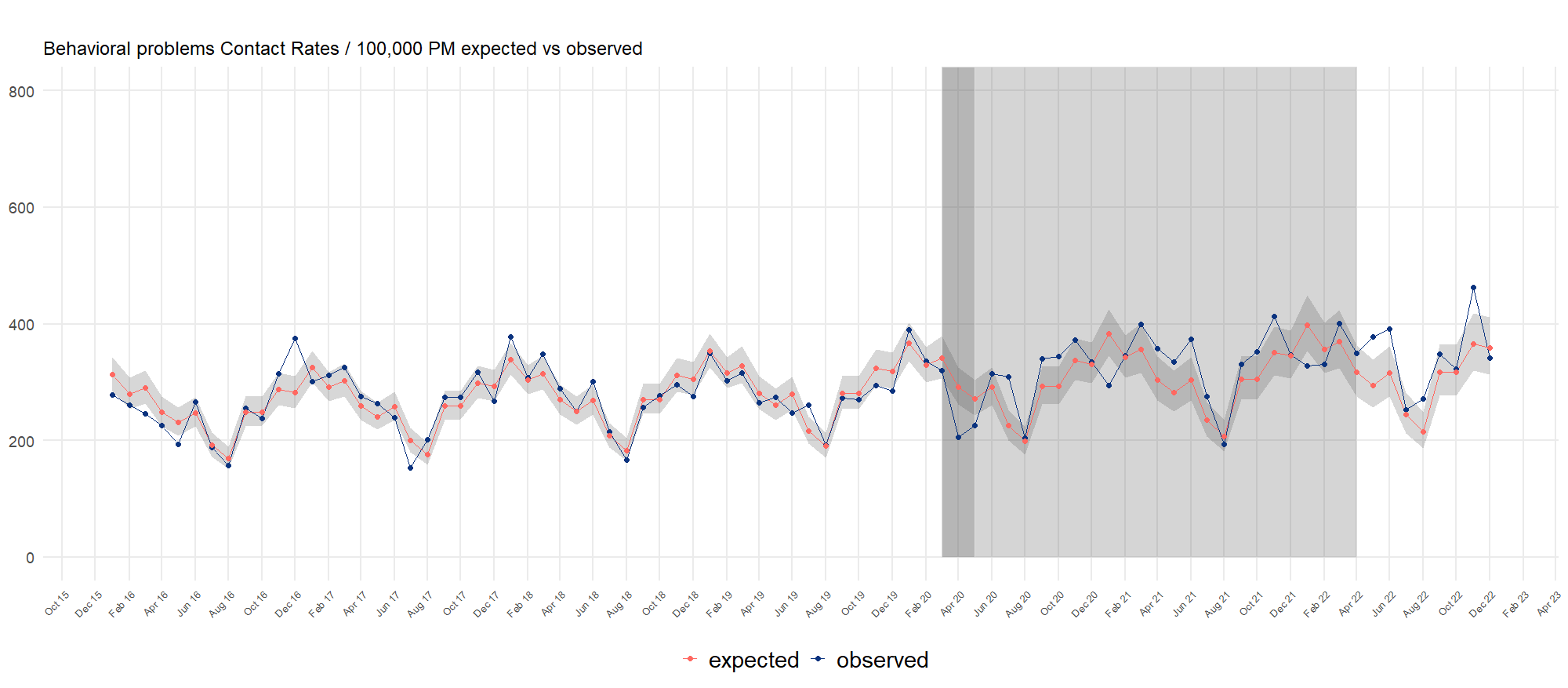

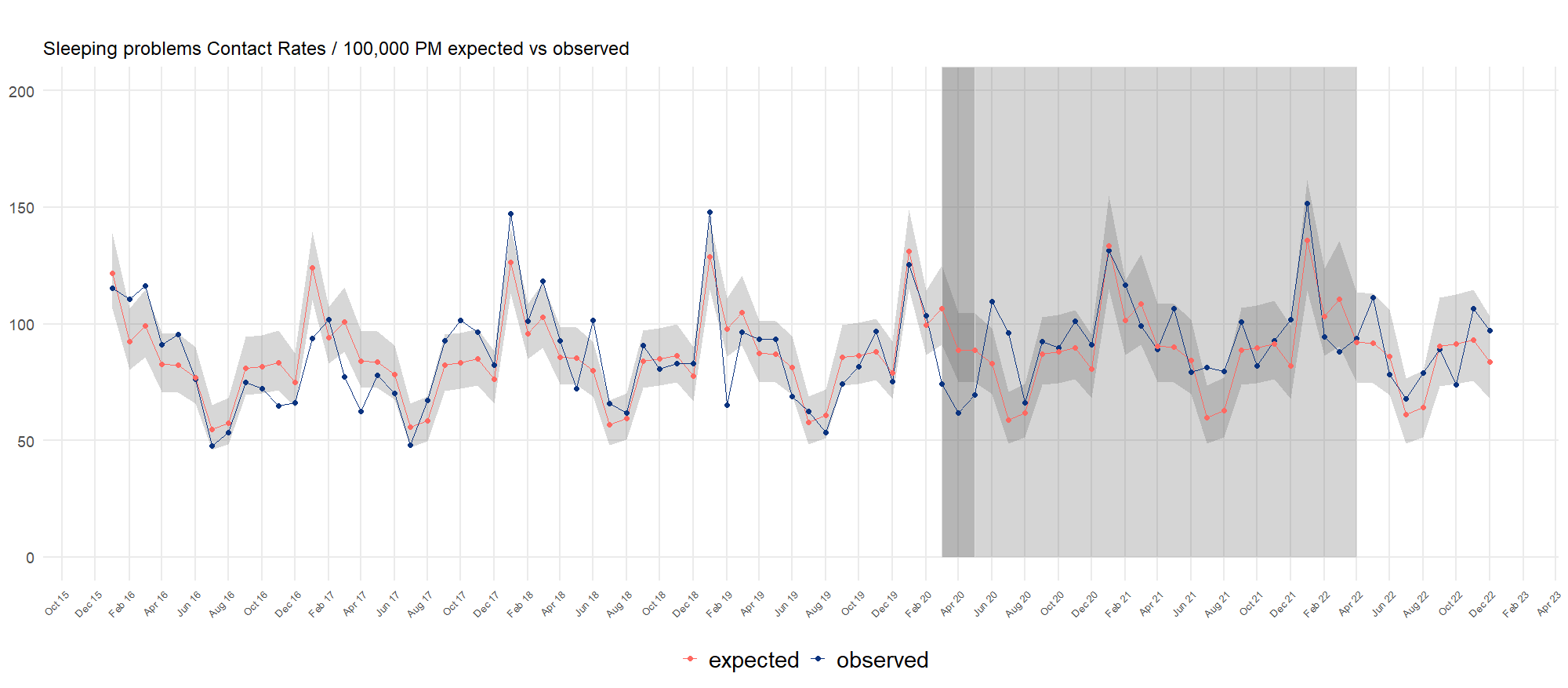


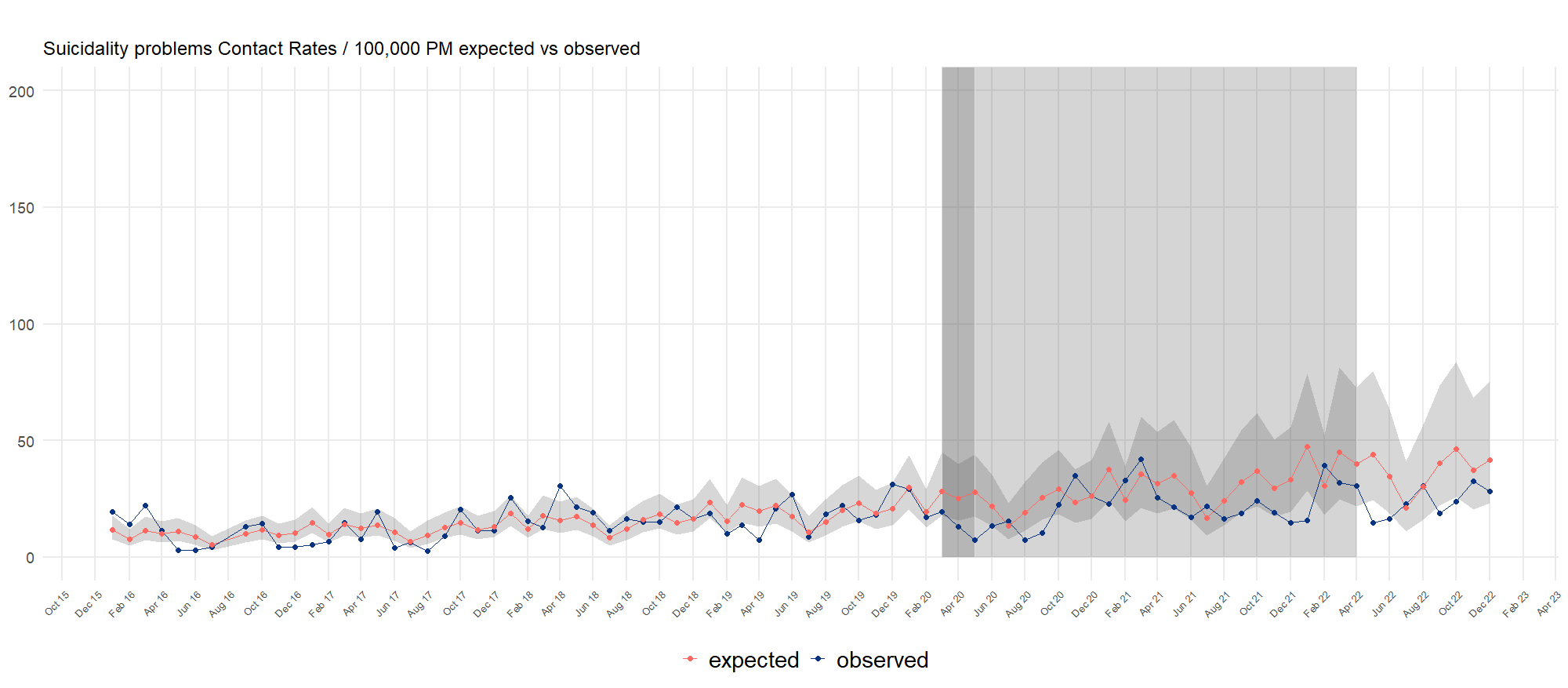

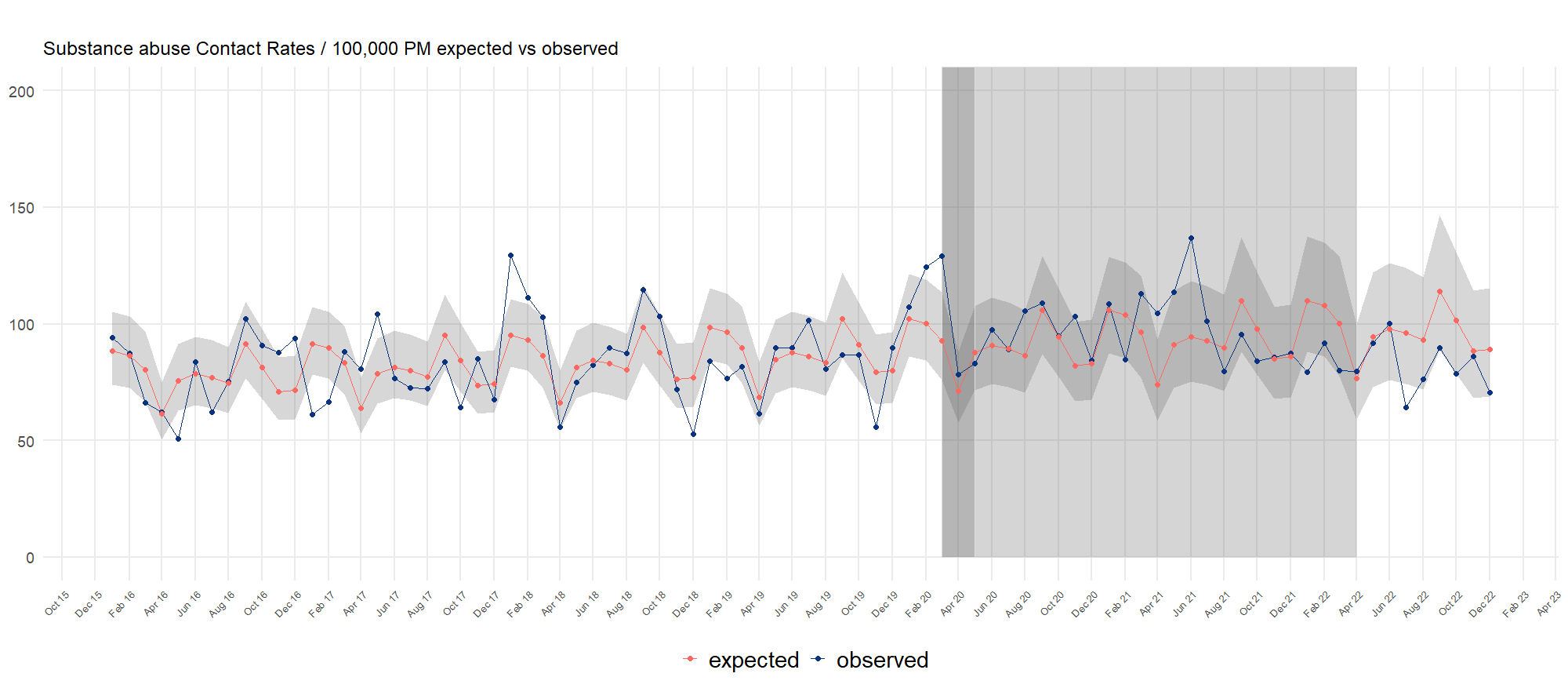


## eFigure 1b modelled monthly consultation rate vs observed consultation rate - continued
